# Deprivation of L-Arginine Induces Oxidative Stress Mediated Apoptosis in *Leishmania donovani* Promastigotes: Contribution of the Polyamine Pathway

**DOI:** 10.1371/journal.pntd.0004373

**Published:** 2016-01-25

**Authors:** Abhishek Mandal, Sushmita Das, Saptarshi Roy, Ayan Kumar Ghosh, Abul Hasan Sardar, Sudha Verma, Savita Saini, Ruby Singh, Kumar Abhishek, Ajay Kumar, Chitra Mandal, Pradeep Das

**Affiliations:** 1 Department of Molecular Biology, Rajendra Memorial Research Institute of Medical Sciences (ICMR), Patna, India; 2 Department of Microbiology, All India Institute of Medical Sciences (AIIMS), Patna, India; 3 Cancer Biology & Inflammatory Disorder Division, CSIR-Indian Institute of Chemical Biology, Kolkata, India; 4 Department of Biotechnology, National Institute of Pharmaceutical Education and Research, Hajipur, India; University of Tokyo, JAPAN

## Abstract

The growth and survival of intracellular parasites depends on the availability of extracellular nutrients. Deprivation of nutrients viz glucose or amino acid alters redox balance in mammalian cells as well as some lower organisms. To further understand the relationship, the mechanistic role of L-arginine in regulation of redox mediated survival of *Leishmania donovani* promastigotes was investigated. L-arginine deprivation from the culture medium was found to inhibit cell growth, reduce proliferation and increase L-arginine uptake. Relative expression of enzymes, involved in L-arginine metabolism, which leads to polyamine and trypanothione biosynthesis, were downregulated causing decreased production of polyamines in L-arginine deprived parasites and cell death. The resultant increase in reactive oxygen species (ROS), due to L-arginine deprivation, correlated with increased NADP+/NADPH ratio, decreased superoxide dismutase (SOD) level, increased lipid peroxidation and reduced thiol content. A deficiency of L-arginine triggered phosphatidyl serine externalization, a change in mitochondrial membrane potential, release of intracellular calcium and cytochrome-c. This finally led to DNA damage in *Leishmania* promastigotes. In summary, the growth and survival of *Leishmania* depends on the availability of extracellular L-arginine. In its absence the parasite undergoes ROS mediated, caspase-independent apoptosis-like cell death. Therefore, L-arginine metabolism pathway could be a probable target for controlling the growth of *Leishmania* parasites and disease pathogenesis.

## Author Summary

*Leishmania donovani*, the causative agent of Indian Visceral Leishmaniasis, resides in the gut of the insect vector and the macrophages of their mammalian host and avail nutrients for survival. Nutrient deprivation such as glucose or amino acid alters redox balance in mammalian cells as well as some lower organisms. However, the role of L-arginine, in regulation of redox balance and *L*. *donovani* survival yet not properly elucidated. In the present study, we found that L-arginine deprivation from the culture medium hinders growth and proliferation of *Leishmania* promastigotes. Starvation of L-arginine downregulates the expression of polyamine biosynthetic and thiol metabolic pathway enzymes leading to decreased production of polyamines in *Leishmania* parasites. Moreover, deprivation of L-arginine alters redox balance in *Leishmania* promastigotes characterized by the concomitant increase in ROS and decreased antioxidant level. Furthermore, L-arginine deprivation triggered phosphatidyl serine externalization, alteration in mitochondrial membrane potential, release of intraellular calcium and cytochrome-c followed by DNA damage. In summary, the growth and survival of *Leishmania* depends on the availability of extracellular L-arginine, in absence of which the parasite undergoes ROS mediated, caspase-independent apoptosis-like cell death. Therefore, targeting L-arginine metabolism pathway could be an alternative approach for controlling *Leishmania* growth and hence disease outcome.

## Introduction

Leishmaniasis, one of the most neglected tropical diseases, is considered as a major global threat spread over 98 countries throughout 5 continents. Among different forms of leishmaniasis, Visceral Leishmaniasis (VL), the most severe one, has a disease burden of 0.2 to 0.4 million cases with a mortality rate of 20,000 to 40,000 reported per year [1]. *Leishmania donovani*, the etiological agent of Indian VL, is an obligatory parasite that harbors inside the sand fly mid-gut and the macrophages of their mammalian host [2, 3]. Maintenance of their niches is crucial for survival and replication of these parasites. Cell death in form of apoptosis carefully controls the population of these parasites which in turn helps in disease progression [4, 5].

Apoptosis or Programmed cell death (PCD) plays a critical role in development and defense response of multicellular organisms [6]. In unicellular trypanosomatids including *Leishmania sp*., apoptosis is a triggered response to different stimuli ranging from heat shock [7–9], reactive oxygen species (ROS) [10–13], antiparasitic drugs [14, 15], starvation [16–18] to antimicrobial peptides [19–20]. Sequentials events encompassing accumulation of reactive oxygen species (ROS), lipid peroxidation, release of intracellular calcium (Ca^2+^) into cytosol, mitochondrial membrane depolarization, externalization of phosphatidyl serine on the outer leaflet, leakage of cytochrome-c into cytosol and induction of caspases resulting in DNA cleavage characterize the onset of apoptosis [21, 22]. Though pathogenic protozoa lacks genes encoding caspases, the involvement of caspase like protease activity has been reported in the regulation of death process of some unicellular organisms [23]. Studies of Lee and co-workers showed that *Leishmania* promastigote undergoes an apoptosis like cell death independent of caspase activities after exposure with antimony [24].

When a cell fails to maintain cellular homeostasis utilizing the total available antioxidant capacity oxidative stress is generated that expedites the process of apoptosis [25]. To protect cells from ROS mediated apoptosis, the parasite must carefully control the level of ROS by upregulating antioxidant defense. Polyamines are one of the crucial molecules that have been shown to exert antioxidant activity [26, 27].

Amino acids in eukaryotes, serve as building blocks in protein biosynthesis and regulates osmotic balance by functioning as osmolytes. In some eukaryotes L-arginine, the precursor for the production of polyamines is not synthesized denovo and is imported to support cellular growth and to protect the cells under diseased conditions [28]. Apoptotic stimuli affect both cellular processes cell proliferation and apoptosis [29]. The role of L-arginine in the regulation of cell survival and apoptosis of some higher eukaryotes have been reported [30, 31]. Piacenza et al. showed the role of L-arginine in modulation of apoptotic death of *T*. *cruzi* epimastigotes [32].

Despite these advances, the precise role of L-arginine in the regulation of redox balance and ROS mediated apoptosis is still unclear in protozoan parasites particularly in *Leishmania sp*. In the present study, we have shown that L-arginine starvation hinders cell growth and proliferation of *Leishmania* parasite. As *Leishmania* parasite lacks the biosynthetic pathway of L-arginine, it upregulates the transport of L-arginine in starved conditions. While investigating the possible reason behind this reduced cell viability, we found that L-arginine deprivation downregulates the production of polyamines that are necessary for the *Leishmania* parasite and alters redox balance characterized by increased ROS level. This results in increased lipid peroxidation and NADP+/NADPH ratio followed by decreased Superoxide dismutase (SOD) activity and thiol levels. Simultaneously, it was observed that arginine starvation induces phosphatidyl serine externalization, mitochondrial membrane depolarization, release of intracellular calcium and cytochrome-c that ultimately damages DNA and promotes apoptosis. Collectively our study reveals, for the first time, the role of L-arginine in redox homeostasis and apoptosis-like cell death in *Leishmania* parasites.

## Materials and Methods

### Culture of parasite

*Leishmania donovani* strain AG83 (MHOM/IN/1983/AG83) originally obtained from an Indian Kala-azar patient was maintained routinely in golden hamsters, as described earlier [33]. In this study, *Leishmania* parasites were grown for 2 generations at 22°C separately in amino acids free and L-arginine free RPMI media (Invitrogen) and supplemented with 10% heat-inactivated dialyzed fetal bovine serum (FBS) (Gibco-BRL), 25 mM HEPES, pH-7.4, 4 mM NaHCO_3_, 100 U/ml of penicillin G-sodium (Sigma-Aldrich) and 100 mg/ml of streptomycin (Sigma-Aldrich) and these parasites were used for our experimental purpose. As per our experimental set up, the exponential phase promastigotes were further grown either in single amino acid (For eg. L-arginine, lysine, glutamine, proline) containing or single amino acid depleted RPMI medium (Invitrogen).

### Cell viability assay

The effect of L-arginine on the viability of *L*. *donovani* promastigotes were analyzed by 3-(4, 5-dimethylthiazol-2-yl)-2, 5-diphenylterazolium bromide (MTT) assay as described previously [34, 35]. *Leishmania* promastigotes of the exponential phase were grown in complete RPMI media (Invitrogen), RPMI media with different concentrations (0–200 mg/L) of L-arginine (Sigma-Aldrich), single amino acid free or supplemented RPMI media as well as complete amino acid free RPMI media for 0–120 hrs. The OD was recorded on an ELISA reader (Multiskan EX; Thermo Fisher Scientific, Waltham, MA) at 570 nm and percent cell viability was determined. The growth of the parasite in absence of L-arginine but presence of L-lysine, glutamine, proline (all from Sigma-Aldrich) or L-ornithine, putrescine, spermidine, spermine (all from Merck-Millipore) as well as in presence of L-arginine was assessed by trypan blue dye exclusion method. Viable cells were quantified by counting the number of non-stained cells. Results were expressed as mean±SD for three independent experiments performed in triplicate.

### BrdU cell proliferation assay

Rate of proliferation of the parasite was analyzed using Cell Proliferation ELISA BrdU colorimetric kit (Roche) as per manufacturer’s instruction as described previously [36]. Briefly ~1×10^5^ parasites/100 μl media were cultured in a 96 well microplate. At different time points, the cells were incubated with 1 μl of 5-Bromo-2-DeoxyUridine (BrdU) labelling agent (final conc. 10 μM) for 5 hr and its uptake was measured at 450 nm on an ELISA reader (Multiskan EX; Thermo Fisher Scientific, Waltham, MA). Results were expressed as mean±SD for three independent experiments performed in triplicate.

### Experimental infection of macrophages with *L*. *donovani* parasites

THP-1cells were treated with 20 nM phorbol 12-myristate 7-acetate (PMA) for 12 hr to become adherent, matured macrophage-like phenotype. Non-adherent cells were removed by washing with RPMI without FBS. The cells were infected with *Leishmania* parasite for 6 hrs at parasite/macrophage multiplicities of 10:1. The unbound parasites were removed by washing with RPMI without FBS followed by additional incubation of upto 24 and 48 hr. The cells were then fixed and stained with May-Gruenwald giemsa and observed under bright field microscope at 100X with oil immersion. Percent infected macrophage was calculated by counting the number of infected cells and parasite load was determined by counting the number of amastigotes per 100 macrophages. Each measurement was performed in triplicate and the data was expressed as mean±SD of three independent experiments.

### Measurement of L-arginine, lysine, glutamine and proline transport

Uptake of L-arginine, lysine, glutamine and proline in *Leishmania* parasites was measured using a protocol as described elsewhere [37] with minor modification. Briefly, after individual incubations, promastigotes (1×10^7^ parasites/ml) were pelleted down, washed twice with cold Earle’s Based Salt Solution (EBSS) containing (in mM) 117 NaCl, 26 NaHCO_3_, 5 KCl, 1.8 CaCl_2_, 1 NaH_2_PO_4_, 0.8 MgSO4, 5.5 glucose and resuspended in 100 μl of [^3^H]-L-arginine (0.5–5 μCi/ml) using stock from 1 mCi/ml (specific activity-51.5 Ci/mmole) or [^3^H]-lysine, [^3^H]-glutamine and [^3^H]-proline (1 μCi/ml) (Perkin-Elmer, Singapore) at 25°C. Uptake was stopped at different times by adding 200 μl of 50 mM ice-cold L-arginine (non-radioactive). Parasites were then washed twice with EBSS (200 μl), lysed in 200 μl lysing solution (0.1% SDS, 100 mM NaOH) and radioactivity was measured by liquid scintillation counter (Tri-Carb 2810 TR, Perkin Elmer, USA). Uptake was expressed as nmol/10^7^ cells/min. Each measurement was performed in triplicate and data was expressed as mean±SD of three independent experiments.

### Semi-quantitative RT-PCR

AD-*Ld* and AS-*Ld* parasites were harvested after 96 hr of incubation. RNA was extracted and c-DNA was prepared from 20μg of total RNA using c-DNA synthesis kit (Invitrogen). The c-DNAs were then amplified by PCR for polyamine biosynthetic and thiol metabolic pathway genes, such as ornithine decarboxylase (ODC), spermidine synthase (SPS), gamma-glutamyl cysteine synthetase (γ-GCS), trypanothione synthetase (TryS), trypanothione reductase (TR), cytoplasmic tryparedoxin (cTXN) and cytoplasmic tryparedoxin peroxidase (CTP) using gene specific primers (Supplementary S1 Table). PCR was performed and analysis was done according to the conditions as described previously [35]. Each measurement was performed in triplicate and data was expressed as mean±SD of three independent experiments.

### Measurement of arginase activity

Arginase activity in L-arginine depleted *Leishmania donovani* (AD-*Ld*) and L-arginine supplemented *Leishmania donovani* (AS-*Ld*) parasite lysates was measured using a micromethod as described previously [38, 39]. Briefly, after individual incubations, cells were lysed in 0.1% Triton X-100 and added with 25 mM Tris-HCl. The cell lysate was then mixed with 10 mM MnCl_2_ and heated for 10 min at 56°C for enzyme activation. Following addition of 0.5 M L-arginine (pH 9.7) to the cell lysate and incubation at 37°C for 15–20 min, the reaction was stopped with H_2_SO_4_ (96%)/H_3_PO_4_ (85%)/H_2_O (1/3/7, v/v/v) mixture. α-isonitrosopropiophenone was added to the mixture and heated at 95°C for 30 min. The urea concentration was then measured spectrophotometrically at 540 nm and total protein concentration was estimated through Bradford method. Each measurement was performed in triplicate and data was expressed as mean±SD of three independent experiments.

### Measurement of polyamines

The level of intracellular polyamines such as putrescine and spermidine in the leishmania parasites grown in arginine depleted and arginine supplemented media (AD-*Ld* and AS-*Ld*) for 24–120 hr were identified and quantified by high performance liquid chromatography (HPLC) using a prederivatization method as described previously [39, 40]. Briefly, after individual incubations, the parasites were harvested, washed with PBS (pH 7.4) and disintegrated with 5% (w/v) trichloroacetic acid for overnight. After centrifugation the supernatants were collected and neutralized with saturated NaHCO3 solution. Dansylation of the mixture was done using dansyl chloride (20 mg/ml) (Merck) in acetone (Merck, HPLC grade) at 50°C for overnight. The polyamines were extracted with toluene (Merck, HPLC grade), and evaporated under nitrogen stream. The residue was dissolved in acetonitrile (Merck, HPLC grade) and analyzed by HPLC on a C18 reverse-phase column (Shimadzu, Japan). Dansylated polyamines were detected and quantified by spectrofluorometric measurement (excitation wavelength 330 nm, emission wavelength 510 nm). The peak areas, retention times were recorded and calculated by a PC Integration Pack Programme. Polyamines were expressed as nmol per 10^7^ promastigotes. There were three replicates in each test and the data were the mean±SD of three independent observations.

### Measurement of reactive oxygen species

*Leishmania* parasites were grown in different invitro culture conditions (For eg. arginine depletion, AD-*Ld*; arginine depletion but L-ornithine supplementation, AD-*Ld*/Orn+; arginine depletion but putrescine supplementation, AD-*Ld*/Put+; arginine depletion but NAC treatment, AD-*Ld*/NAC+ and arginine supplementation, AS-*Ld*) for 0–120 hr. After individual incubations were over, the cells were harvested and the level of intracellular ROS was monitored by staining the cells with the oxidative fluorescent dye 2', 7'-dichlorodihydrofluorescein diacetate (H_2_DCFDA) (0.4 μM) (Sigma) for 15 min at 37°C and then analyzed using LS-55 spectrofluorometer (Perkin Elmer, USA) as described previously [41]. Absorbance and emission wavelength were 504 and 529 nm respectively. There were three replicates in each test, and the data were the mean±SD of three independent observations.

### Measurement of total fluorescent lipid peroxidation product

*Leishmania* parasites were grown in different invitro culture media for 0–120 hr as mentioned earlier. After individual incubations, AD-*Ld*, AD-*Ld*/Orn+, AD-*Ld*/Put+, AD-*Ld*/NAC+ and AS-*Ld* parasites were harvested, washed with 1X PBS and dissolved in 15% SDS-PBS solution. The total fluorescent lipid peroxidation products were estimated spectrofluorometrically with excitation at 360 nm and emission at 430 nm. The results were expressed as relative fluorescence units with respect to quinine sulphate (1 mg/ml in 0.5 M H_2_SO_4_) [42, 43]. The data were mean±SD for three independent experiments performed in triplicate.

### Determination of reduced thiol content

The total intracellular reduced thiol level was measured in deproteinized cell extracts from *Leishmania* parasites grown for 72–120 hr in different invitro culture conditions (AD-*Ld*, AD-*Ld*/Orn+, AD-*Ld*/Put+, AD-*Ld*/NAC+ and AS-*Ld*) by the method as described elsewhere [44]. Briefly, the cells were harvested, washed with a buffer [0.14 M Na_3_PO_4_, 0.14 M K_3_PO_4_, 0.14 M NaCl, and 3 mM KCl, pH-7.4] and suspended in 25% trichloroacetic acid. The denatured protein and cell debris were removed by centrifugation. Thiol content in the supernatant was determined with 0.6 mM 5, 5’-dithio-bis(2-nitrobenzoic acid) (DTNB, Ellman’s reagent) in 0.2 M Na_3_PO_4_ buffer, pH 8.0. DTNB derivatives of thiols were estimated spectrophotometrically at 412 nm. There were three replicates in each test, and the data were expressed as mean±SD of three independent observations.

### Determination of SOD activity

SOD activity was measured by using SOD assay kit (SOD assay kit, Calbiochem) as per manufacturer’s instruction. Briefly, after individual incubations, the parasites, as mentioned earlier, grown in different invitro culture conditions (AD-*Ld*, AD-*Ld*/Orn+, AD-*Ld*/Put+, AD-*Ld*/NAC+ and AS-*Ld*) for 72–120 hr, were pelletted down, homogenized in cold 20 mM HEPES buffer (pH-7.2, containing 1 mM EGTA, 210 mM mannitol and 70 mM sucrose) and the supernatants were collected for assay. To 10 μl of supernatant, 200 μl of diluted radical detector was added followed by addition of 20 μl of diluted xanthine oxidase and incubation for 20 minutes. The absorbance was measured at 450 nm. One unit of SOD was defined as the amount of enzyme required to exhibit 50% dismutation of the superoxide radical. Data were expressed as mean±SD for three independent experiments performed in triplicate.

### Measurement of NADP+/NADPH ratio

NADP+/NADPH ratio for the AD-*Ld*, AD-*Ld*/Orn+, AD-*Ld*/Put+, AD-*Ld*/NAC+ and AS-*Ld* parasites were assayed spectrophotometrically using NADP/NADPH Quantification Kit (Sigma) as per manufacturer’s instruction as described [45]. The reduced coenzyme was measured spectrophotometrically at 450 nm. Results were mean±SD for three independent experiments performed in triplicate.

### Annexin-V binding assay

Annexin-V-FITC staining was performed by the method as described previously with Annexin-V-FLUOS staining kit (Roche) as per manufacturer’s instructions [46]. Briefly, after individual incubations, the *Leishmania* parasites grown in different invitro culture conditions (AD-*Ld*, AD-*Ld*/Orn+, AD-*Ld*/Put+, AD-*Ld*/NAC+ and AS-*Ld*) for 72–120 hr were harvested, washed twice in PBS (pH 7.2), resuspended in HEPES buffer followed by addition of Annexin V-FITC and PI. The cells were then incubated for 15 min in the dark at 25°C and acquired on BD FACS Aria II flow cytometer followed by analysis with BD FACS Diva software. Results were expressed as mean±SD for three independent experiments performed in triplicate.

### Measurement of mitochondrial membrane potential (Δψm)

Change in mitochondrial membrane potential (Δψm) in *Leishmania* promastigotes was estimated using JC-1 dye as described previously [46]. Briefly, after 72–120 hr of incubation, *Leishmania* parasites grown in different invitro conditions (For eg. arginine depletion, AD-*Ld*; arginine depletion but L-ornithine supplementation, AD-*Ld*/Orn+; arginine depletion but putrescine supplementation, AD-*Ld*/Put+; arginine depletion but NAC treatment, AD-*Ld*/NAC+; L-arginine supplementation, AS-*Ld* and L-arginine supplementation but FCCP treatment, AS-*Ld*/FCCP+) were harvested and incubated with 10 μM JC-1 for 10 min at 37°C. The cells were washed, resuspended in media followed by fluorescence measurement using LS-55 spectrofluorometer (Perkin Elmer, USA). Relative Δψm value was expressed as ratio of the reading at 590 nm to the reading at 530 nm. Results were expressed as mean±SD from three independent experiments performed in triplicate.

### Measurement of intracellular calcium (Ca^2+^) level

Intracellular Calcium concentration in AD-*Ld*, AD-*Ld*/Orn+, AD-*Ld*/Put+, AD-*Ld*/NAC+, AD-*Ld*/EGTA+ and AS-*Ld* parasites was measured following the protocol and using the fluorescent probe Fura-2-AM as described previously [43]. Each measurement was performed in triplicate and the data were expressed as mean±SD for three independent experiments.

### TUNEL assay

The occurrence of DNA nicking generated during apoptosis was detected by the Terminal deoxynucleotidyl transferase dUTP nick end labelling (TUNEL) assay as described previously using In situ Cell Death Detection kit, Fluorescein (Roche) as per manufacturer’s instruction [47]. Briefly, AD-*Ld*, AD-*Ld*/Orn+, AD-*Ld*/Put+, AD-*Ld*/NAC+ and AS-*Ld* parasites were harvested as mentioned earlier and dissolved in 100 μl of 1X PBS followed by addition of 50 μl of Fixation and Permeabilizing solution and incubation at 4°C for 15 min. The cells were pelleted down, washed with 1X PBS, resuspended in 50 μl of TUNEL mixture and incubated at 22°C BOD incubator for 1 hr. Finally, the cells were washed and resuspended in PBS and analyzed by flow cytometry (BD FACS Aria II). Each measurement was performed in triplicate and the data were expressed as mean±SD for three independent experiments.

### Cell death detection ELISA

DNA fragmentation was assessed using Cell Death Detection ELISA plus kit (Roche) as per manufacturer’s instruction. Briefly, after individual incubations, AD-*Ld*, AD-*Ld*/Orn+, AD-*Ld*/Put+, AD-*Ld*/NAC+ and AS-*Ld* parasites were harvested, lysed and reacted with biotin-coupled mouse monoclonal anti-histone antibody. The complex was detected by peroxidase-conjugated mouse monoclonal anti-DNA antibody and ABTS [2, 2’-azino-bis(3-ethylbenzthiazoline-6-sulphonic acid)] as a developing reagent to quantify the cytoplasmic nucleosomes generated due to DNA fragmentation. Absorbance was measured in a Thermo Multiskan EX plate reader at 405 nm and the results were expressed as relative percentages. Each measurement was performed in triplicate and the data were expressed as mean±SD for three independent experiments.

### Immunoblot analysis

*Leishmania* parasites grown in different invitro conditions (For eg. arginine depletion, AD-*Ld*; arginine depletion but L-ornithine supplementation, AD-*Ld*/Orn+; arginine depletion but putrescine supplementation, AD-*Ld*/Put+; arginine depletion but NAC treatment, AD-*Ld*/NAC+; L-arginine supplementation, AS-*Ld* and L-arginine supplementation but Camptothecin-B treatment, AS-*Ld*/CPT+) were harvested after 96 hrs of incubation and washed twice with 1X PBS, suspended in cell fractionation buffer (ApoAlert cell fractionation kit, BD), homogenized and cytosolic & mitochondrial fractions were separated by following the manufacturer’s instructions (Clontech, Palo Alto, CA, USA) [47]. For confirmation of the semi-quantitative RT-PCR data, 50μg cytosolic fractions collected from AD-*Ld* and AS-*Ld* parasites were run on 12% SDS-polyacrylamide gel, immunoblotted and probed with rabbit polyclonal anti-TryS (1:5000), anti-TR (1:2000) and anti-CTP (1:5000) antibodies. For cytochrome-c detection, the cytosolic fraction from all the treated parasites were immunoblotted and probed with the rabbit polyclonal anti-cytochrome-c antibody (1:1000). Horseradish peroxidase-conjugated secondary antibody (1:10000) was used for all the cases and alpha-tubulin (α-Tub) was used as an endogenous control. The protein band was visualized by enhanced chemiluminescence reaction and expressed as fold change.

### Assay of caspase activity

To determine the caspase activity (caspase—1, 3, 5, 8 and 9) of *Leishmania* parasites, a fluorogenic homogeneous caspase assay was performed using the Caspase Family Fluorometric Substrate Set plus kit (Bio-Vision) according to manufacturer’s instructions. The fluorometric measurement was recorded with a LS-55 spectrofluorometer (Perkin Elmer, USA) with excitation at 400 nm and emission at 505 nm. Each measurement was performed in triplicate and data was expressed as mean±SD of three independent experiments.

### Statistical analysis

Statistical analysis was performed using GraphPad Prism Program (Version 6.0, GraphPad Software, USA). All results were shown as mean±SD. Statistically significant differences were determined using students t-test. P values equal or below 0.05 were considered significant.

## Results

### L-arginine is crucial for growth of *L*. *donovani* promastigotes

**Fig 1:**
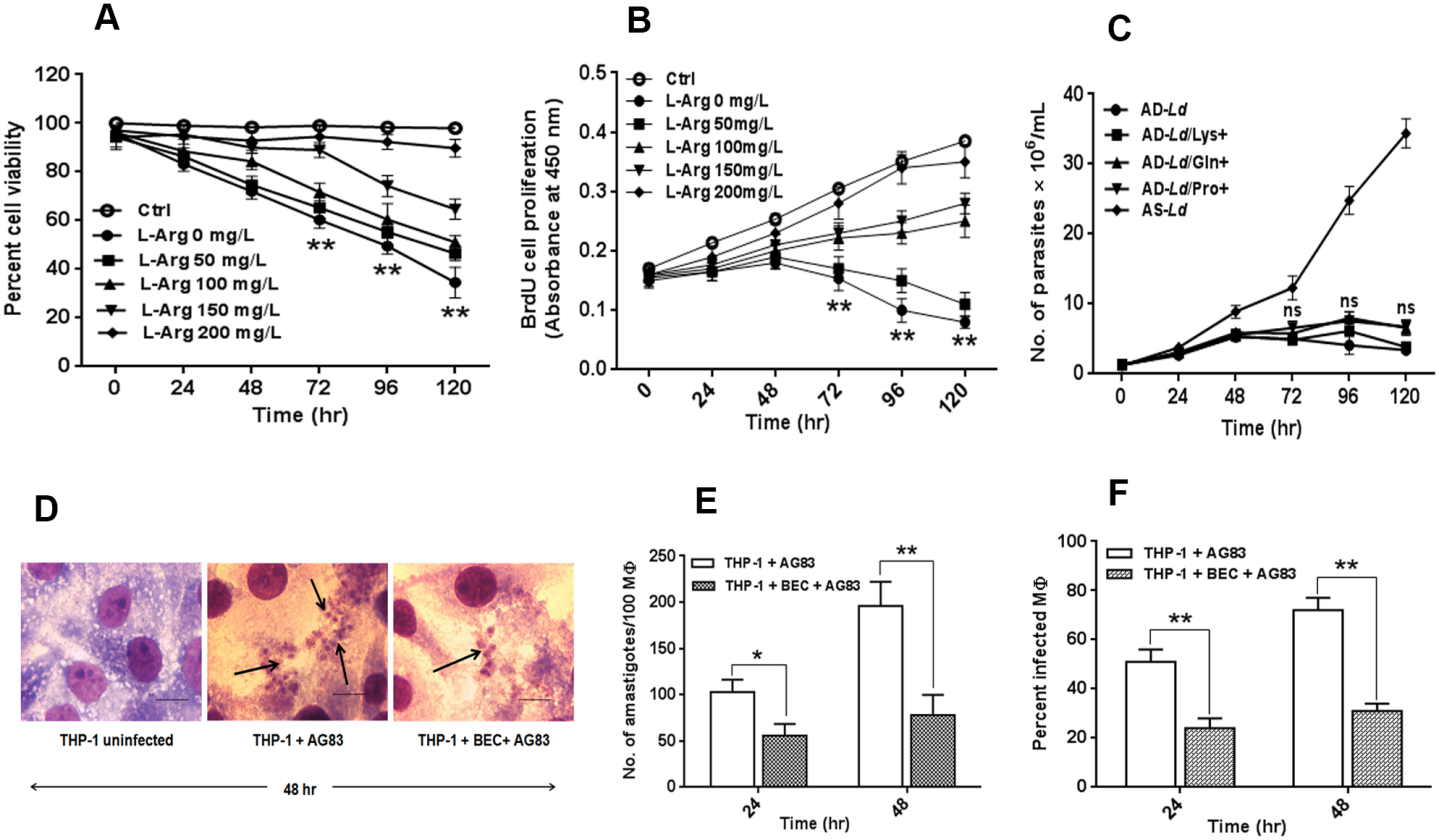
L-arginine deprivation reduces cell viability, controls growth and proliferation of *Leishmania* parasite. (A-C) *L*. *donovani* promastigotes were grown in normal RPMI (Ctrl or AS-*Ld*), L-arginine depleted RPMI media (L-Arg 0 mg/L or AD-*Ld*), supplemented with 50–200 mg/L of L-arginine or 200 mg/L lysine (AD-*Ld*/Lys+), glutamine (AD-*Ld*/Gln+) and proline (AD-*Ld*/Pro+) as described in “Materials and methods”. The percent cell viability of the parasite was determined by MTT assay (A). The rate of parasite proliferation was determined by BrdU colorimetric cell proliferation ELISA (B). The growth rate of the parasite was determined by counting the number of viable cells using trypan blue dye exclusion method (C). The data represents mean±SD of triplicate determinations and are representative of three independent experiments. *, P<0.05 (Student’s t test), **, P<0.001 compared to control (or AD-*Ld* as applicable). ns, non-significant. (D-F) THP-1 macrophages were either uninfected or infected with *Leishmania* parasites in absence or presence of BEC (200μM), an inhibitor of L-arginine utilizing enzyme, arginase for 24 hr and 48 hr. Intracellular parasites were visualized by staining the cells with giemsa followed by optical microscopy at 100X oil immersion (D). The horizontal bar indicates 5μm size. The parasite load was measured by counting the number of intracellular amastigotes per 100 macrophages (MΦ) (E). The rate of infection was analyzed by counting the percent infected macrophages (F). The data represent mean±SD of triplicate determinations and are representative of three independent experiments.*, P<0.05 (Student’s t test), **, P<0.001 compared to control (or infected macrophage as applicable). ns, non-significant

In this study, we used RPMI-1640 without L-arginine media and supplemented with upto 200 mg/L (1.149 mM) of L-arginine to characterize the role of L-arginine in the growth and survival of *L*. *donovani* promastigotes [48, 49]. Results showed that the percent cell viability decreased with increasing time interval. A significant difference (~1.65 fold, ~2.0 fold and ~2.8 fold) in reduction in cell viability was observed at 72 hr, 96 hr and 120 hr respectively for L-arginine deprived parasites compared to control parasites (Fig 1A).

The viability of the *Leishmania* parasite in single amino acid free (for eg. L-arginine, lysine, glutamine and proline) as well as complete amino acid free (AA-) RPMI media were also analyzed. It was found that the viability of the *Leishmania* parasite is significantly reduced in amino acid deprived media and L-arginine deprived media compared to normal RPMI media (ctrl). However, the reduction in cell viability observed in lysine, glutamine or proline free media was insignificant (S1A Fig). Simultaneously, cell viability in amino acid free but single amino acid supplemented (200 mg/L) media was also investigated. The result indicates that single amino acid L-arginine supplementation restored the cell viability upto ~80% whereas other amino acids failed to restore the growth (S1B Fig).

BrdU cell proliferation assay was conducted and found in all cases, that the rate of cell proliferation increased with increasing time intervals up to 48 hr. However, the rate of cell proliferation decreased from the 48 hr time point onwards. Despite L-arginine supplemented parasites (L-Arg 100–200 mg/L) showing considerably increased proliferation up to 120 hr, the rate of cell proliferation decreased gradually in L-arginine deprived parasites (L-Arg 50 mg/L and L-Arg 0 mg/L) up to 120 hr (Fig 1B). The minimum *Leishmania* parasites proliferation rate was observed when grown in L-arginine depleted media. The decrease in rate of cell proliferation was found to be ~2.0 fold, ~3.5 fold and ~4.8 fold at 72 hr, 96 hr and 120 hr respectively for L-arginine deprived parasites compared to parasites grown in normal RPMI media.

Experiments, to further understand the inhibition of growth, induced by L-arginine deprivation, were conducted to investigate whether supplementing equal amount of other amino acids such as lysine, glutamine and proline (200 mg/L) to the culture medium could reverse the effect. The addition of these amino acids did not produce any noticeable significant changes in the growth patterns of arginine depleted *Leishmania donovani* (AD-*Ld*) (Fig 1C).

To confirm the importance of L-arginine in the growth of the *Leishmania* parasite inside macrophages, we infected THP-1, a human monocytic macrophage like cell line with *Leishmania* parasite in the presence or absence of S-(2-Boronoethyl)-L-cysteine (BEC), an inhibitor of L-arginine-utilizing enzyme, arginase for 24 hr and 48 hrs. The sole objective behind the use of BEC was to prevent the macrophages from utilizing the available L-arginine in the extracellular milieu. This was to create an L-arginine deprived condition for the macrophages and to examine the resulting effect on parasite survival by determining the parasitic load and percent infectivity. It was found that the parasite load (Number of amastigotes per 100 macrophages) decreased by ~1.8 fold and ~2.5 fold at 24 hr and 48 hr respectively in presence of BEC (Fig 1D and 1E) when compared to control. Simultaneously, percent infectivity also decreased by ~2.1 fold and ~2.3 fold respectively at 24 hr and 48 hr in presence of BEC (Fig 1F). Therefore, our results indicate that inhibition of arginase or limited L-arginine availability led to reduced level of infection.

### L-arginine starvation in *Leishmania* parasite up-regulates its transport in time and concentration dependent manner

**Fig 2:**
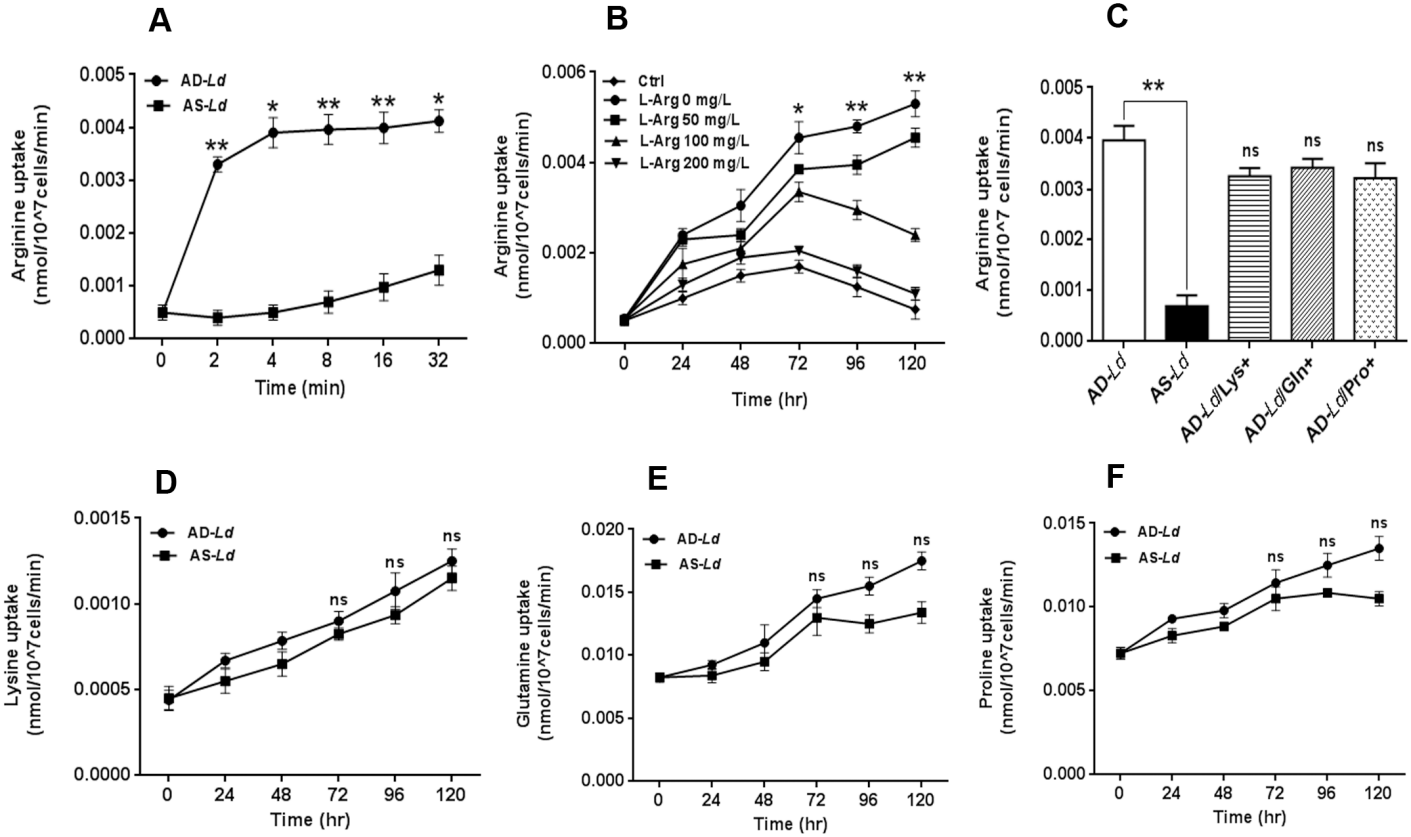
L-arginine deprivation in *Leishmania* parasite upregulates its transport in time and concentration dependent manner. (A) *Leishmania* parasites were grown in L-arginine depleted (AD-*Ld*) and L-arginine supplemented (AS-*Ld*) RPMI media for 96 hr, incubated with 1μCi/ml of [^3^H] L-arginine uptake solution for 0–32 minutes and rate of uptake was measured. (B-C) *Leishmania* promastigotes were grown in normal RPMI (Ctrl or AS-*Ld*), L-arginine depleted RPMI media (L-Arg 0 mg/L or AD-*Ld*) as well as supplemented with 50–200 mg/L of L-arginine or 200 mg/L lysine (AD-*Ld*/Lys+), glutamine (AD-*Ld*/Gln+) and proline (AD-*Ld*/Pro+) as described in “Materials and methods” and incubated with 1μCi/ml of [^3^H] L-arginine uptake solution for 8 minutes. The rate of L-arginine transport was measured by counting the incorporation of [^3^H] L-arginine in liquid scintillation counter (A-C). The data represents mean±SD of triplicate determinations and are representative of three independent experiments.*, P<0.05 (Student’s t test), **, P<0.005 compared to AS-*Ld* or control (or AD-*Ld* as applicable) parasite as applicable. ns, non-significant. (D-F) *Leishmania* parasites were grown in L-arginine depleted (AD-*Ld*) and L-arginine supplemented (AS-*Ld*) RPMI media for 96 hr and incubated with 1μCi/ml of [^3^H] lysine, [^3^H] glutamine and [^3^H] proline uptake solution for 8 minutes. The rate of transport of lysine (D), glutamine (E) and proline (F) was measured by counting the radioactivity incorporation in liquid scintillation counter (D-F). The data represents mean±SD of triplicate determinations and are representative of three independent experiments.*, P<0.05 (Student’s t test), **, P<0.001 compared to AS-*Ld* parasite as applicable. ns, non-significant.

The importance of L-arginine was further characterized by measuring the radioactive L-arginine transport in *Leishmania* parasite. Firstly, experiments were performed to determine a concentration kinetics of radioactive L-arginine (0–5 μCi/ml) using stock from 1 mCi/ml (specific activity-51.5 Ci/mmole) and measure its transport in *Leishmania* parasites grown in L-arginine deprived or supplemented media. Results indicated that the uptake of L-arginine increases with increasing extracellular radioactive L-arginine concentration (S2 Fig). The rate of increase in transport was found to be comparatively higher in arginine depleted *Leishmania donovani* (AD-*Ld*) compared to arginine supplemented *Leishmania donovani* (AS-*Ld*). In both cases, measurable amounts of L-arginine uptake was observed when we used 1 μCi/ml of extracellular ^3^H L-arginine (S2 Fig). To understand how quickly L-arginine is taken up by the *Leishmania* parasite, we performed a time kinetics of L-arginine transport according to previously described methods [37]. We treated AD-*Ld* and AS-*Ld* parasites with ^3^H L-arginine for 0–32 minutes and found that within 4 minutes of incubation the uptake increases at a very high rate and afterwards reaches a plateau in case of AD-*Ld* parasites. In comparison, AS-*Ld* parasites uptake still occurred but with very slow rate (Fig 2A). This result indicated that the rate of L-arginine uptake depended on the time of incubation and most of the L-arginine was uptaken by the *Leishmania* parasites within 8 minutes. Therefore, based on the results obtained we selected 1 μCi/ml dose of L-arginine for 8 minutes incubation in all the subsequent experiments.

To further understand the effect of L-arginine starvation on the transport of L-arginine in a broader time interval, we performed transport measurement experiments in *Leishmania* parasites grown in L-arginine depleted media supplemented with different conc. of L-arginine (0–200 mg/L) for different time intervals (0–120 hr). It was found that L-arginine transport increased gradually with increasing time of incubation. AD-*Ld* parasites were found to uptake L-arginine at a very high rate in comparison to AS-*Ld* parasites. Significant difference in L-arginine uptake was observed 72 hours onwards. ~2.7 fold, ~3.8 fold and ~7.1 fold increase in L-arginine uptake was observed for AD-*Ld* parasites compared with AS-*Ld* parasites at 72 hr, 96 hr and 120 hr respectively (Fig 2B). The transport of L-arginine in the presence of other amino acids such as lysine, glutamine and proline was also measured (Fig 2C). However, no difference in uptake was observed between AD-*Ld* and AD-*Ld*/Lys+ or AD-*Ld*/Gln+ or AD-*Ld*/Pro+ parasites. On the other hand, when we measured the transport of lysine (Fig 2D), glutamine (Fig 2E) and proline (Fig 2F) in AD-*Ld* and AS-*Ld* parasites, no difference in uptake was observed. Taken together, these results indicate that L-arginine deprivation in *Leishmania* parasite specifically up-regulates its transport in time and concentration dependent manner.

### L-arginine deprivation reduces expression of polyamine biosynthetic and thiol metabolic pathway enzymes and polyamine production

**Fig 3:**
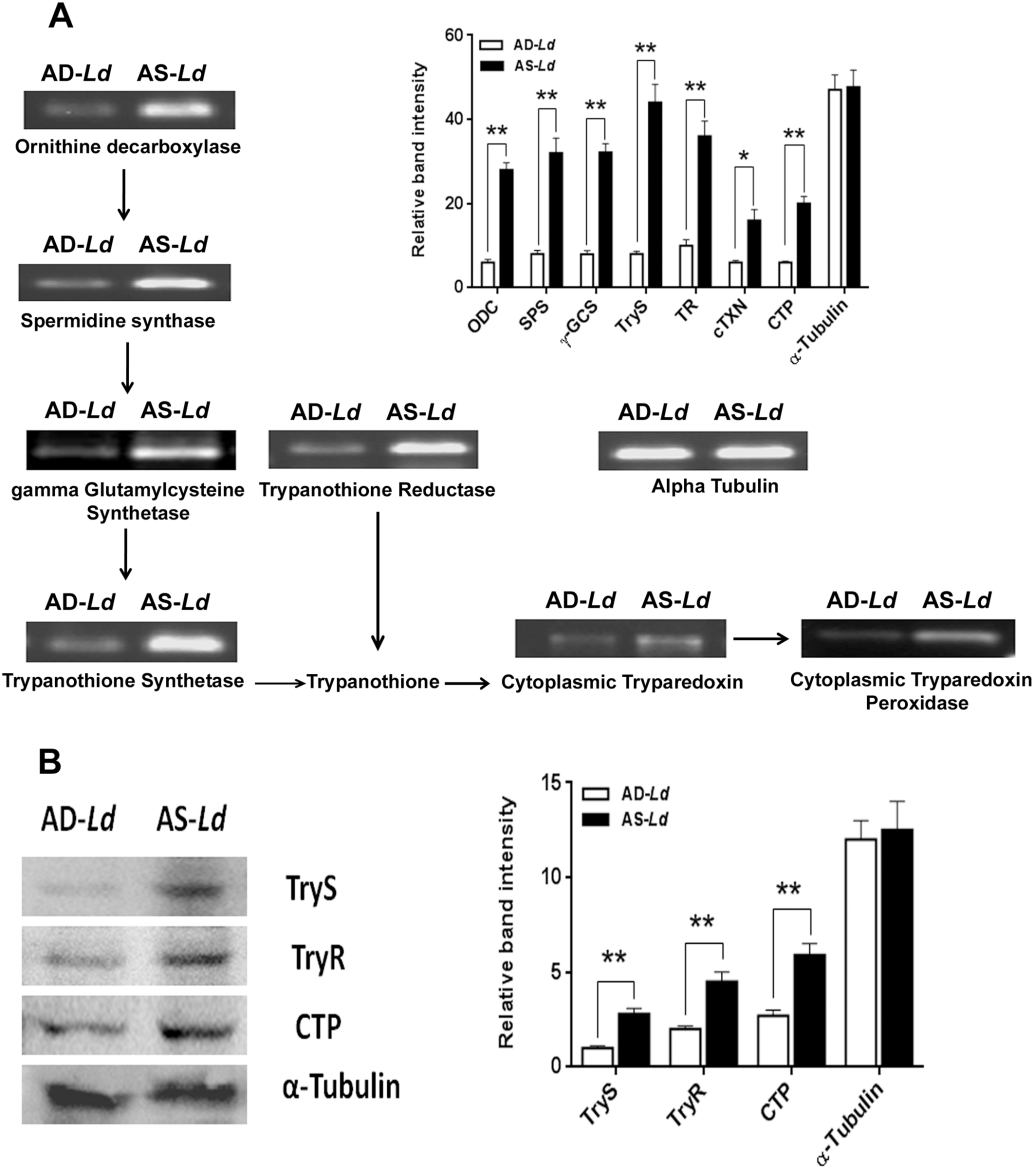
L-arginine deprivation downregulates the expression of polyamine biosynthetic and thiol-metabolic pathway enzymes. (A-B) *Leishmania* parasites were grown in L-arginine depleted (AD-*Ld*) and L-arginine supplemented (AS-*Ld*) RPMI media for 96 hr. (A) The cells were harvested, total RNA was extracted, c-DNA was prepared and expression of ornithine decarboxylase (ODC), spermidine synthase (SPS), γ-glutamyl cysteine synthetase (γ-GCS), trypanothione synthetase (TryS), trypanothione reductase (TR), cytoplasmic tryparedoxin (cTXN), cytoplasmic tryparedoxin peroxidase (CTP) and alpha-tubulin were analyzed by semi-QRT PCR using primers for the coding regions. The PCR products were run in ethidium bromide-stained 1.5% agarose gel and photographed in Gel documentation system. (B) The cells were harvested and cytoplasmic fractions were separated by the procedure as described in “Materials and method”. Cytosolic fractions collected from AD-*Ld* and AS-*Ld* parasites were run on SDS-polyacrylamide gel, immunoblotted and probed with rabbit polyclonal anti-leishmania TryS, TR, CTP and alpha-tubulin antibodies. The relative expression of the enzymes both at transcriptional and translational level were analyzed by densitometry (A-B). The data represent mean±SD of triplicate determinations and are representative of three independent experiments.*, P<0.05 (Student’s t test), **, P<0.001 compared to AS-*Ld* as applicable.

The polyamine biosynthetic and thiol metabolic pathway enzymes such as ODC, SPS, γ-GCS, TryS, TR, c-TXN and CTP are involved in the synthesis polyamines, trypanothione and tryparedoxin utilizing L-arginine as a primary substrate. Polyamines play a crucial role in the growth and survival of trypanosomatids including *Leishmania* parasites. The effect of L-arginine starvation on the transcripts of these enzymes were also determined. A significant decrease in transcript abundance of ODC (~4.7 fold), SPS (~4.0 fold), γ-GCS (~4.0 fold), TryS (~5.5 fold), TR (~3.6 fold), cTXN (~2.7 fold) and CTP (~3.3 fold) was observed for AD-*Ld* parasites as assessed by semi-quantitative RT PCR (Fig 3A).

The expression of some of the enzymes of the polyamine and thiol metabolic pathway (For eg. TryS, TR and CTP) was also evaluated at the translational level by immunoblot analysis. A significant decrease in protein level of Trys (~2.8 fold), TR (~2.3 fold) and CTP (~2.2 fold) was observed for AD-*Ld* parasites compared to AS-*Ld* parasites (Fig 3B).

**Fig 4:**
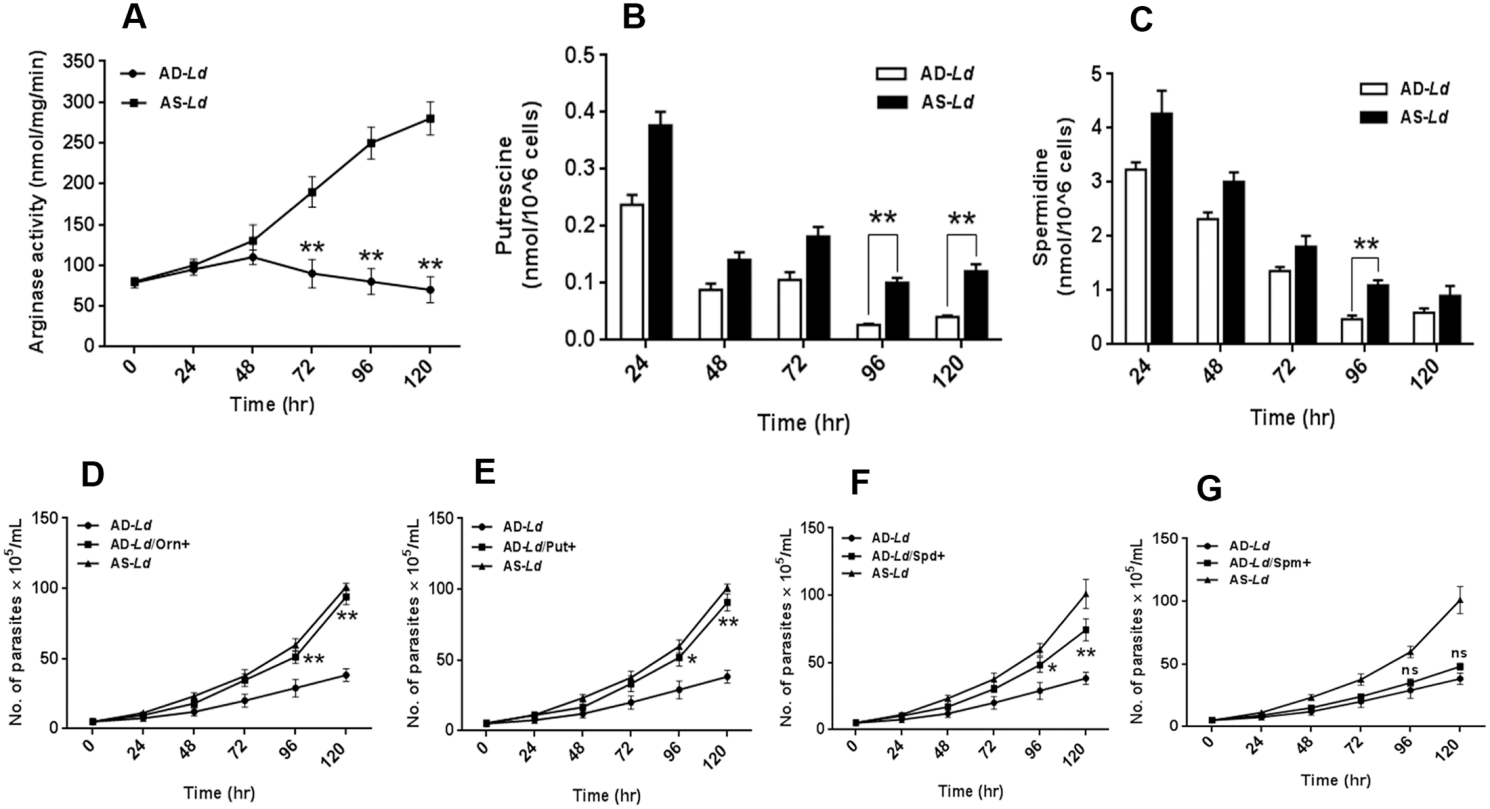
L-arginine deprivation reduces arginase activity and production of polyamines that supports the growth of *Leishmania*. (A-C) *Leishmania* parasites were grown in L-arginine depleted (AD-*Ld*) and L-arginine supplemented (AS-*Ld*) RPMI media for 0–120 hr. Leishmanial arginase activity was measured spectrophotometrically following procedures as described in “Materials and methods”. The protein content was measured by Bradford method and the enzyme activity was expressed as nmol/mg/min (A). (B-C) The polyamines, putrescine and spermidine were extracted by TCA precipitation followed by dansyl chloride derivatization, separation by reverse phase HPLC as described in “Materials and methods”. Dansylated putrescine (B) and dansylated spermidine (C) were quantified by fluorescence spectrophotometry. The data represents mean±SD of triplicate determinations and are representative of three independent experiments.*, P<0.05 (Student’s t test), **, P<0.001 compared to AD-*Ld* or AS-*Ld* parasite as applicable. ns, non-significant. (D-G) *Leishmania* parasites were grown in L-arginine depleted (AD-*Ld*), L-arginine supplemented (AS-*Ld*), L-arginine depleted but supplemented with 200 mg/L of L-ornithine (AD-*Ld*/Orn+) (D), putrescine (AD-*Ld*/Put+) (E), spermidine (AD-*Ld*/Spd+) (F) and spermine (AD-*Ld*/Spm+) (G) media for 0–120 hr. The growth rate of the parasites under different conditions was determined by microscopic counting of viable cells. The data represents mean±SD of triplicate determinations and are representative of three independent experiments.*, P<0.05 (Student’s t test), **, P<0.001 compared to AD-*Ld* or AS-*Ld* parasite as applicable. ns, non-significant.

To study the effect of L-arginine availability in regulation of arginase, the first enzyme of arginine metabolism, the activity of the enzyme was also measured. Fig 4A shows the changes in arginase activity in arginine supplemented *Leishmania donovani* (AS-*Ld*) and arginine depleted *Leishmania donovani* (AD-*Ld*) parasites for 0–120 hr. Differences in arginase activity were apparent from 72 hours post incubation (Fig 4A). ~2.1 fold, ~3.1 fold and ~4.0 fold decrease in arginase activity was observed for AD-*Ld* parasites when compared with AS-*Ld* parasites at 72 hr, 96 hr and 120 hr respectively.

Subsequently, the level of intracellular polyamines (putrescine and spermidine) were quantified by HPLC. Interestingly, we found that at all the time intervals the level of polyamines were lower in arginine depleted *Leishmania donovani* (AD-*Ld*) parasites compared to arginine supplemented *Leishmania donovani* (AS-*Ld*) parasites. At 96 hr and 120 hour a marked difference in putrescine level (~3.7 and ~2.9 fold) was observed (Fig 4B), whereas the difference in spermidine content was found to be very less in AD-*Ld* and AS-*Ld* parasites (~1.7 and ~1.3 fold) (Fig 4C). However, the concentration of both polyamines decreased with increasing time in all cases.

### L-ornithine and polyamine supplementation partially restores the growth of *Leishmania* parasite

We also determined the growth of the parasite in L-arginine depleted media and L-arginine depleted but supplemented with 200 mg/L of L-ornithine, putrescine, spermidine and spermine, in order to confirm that the inhibition of growth of *Leishmania* parasite in L-arginine deprived media is due to polyamine depletion. We have chosen this concentration of L-ornithine and polyamines because 200 mg/L of L-arginine was the optimum concentration required for *Leishmania* growth. L-ornithine and putrescine supplementation was found to almost completely restored the growth of the parasite (Fig 4D and 4E). In comparison, spermidine restored growth of the parasite to lesser extent (Fig 4F) and spermine had very negligible effects on restoration of growth (Fig 4G).

### L-arginine deprivation alters redox balance in *L*. *donovani* promastigotes

**Fig 5:**
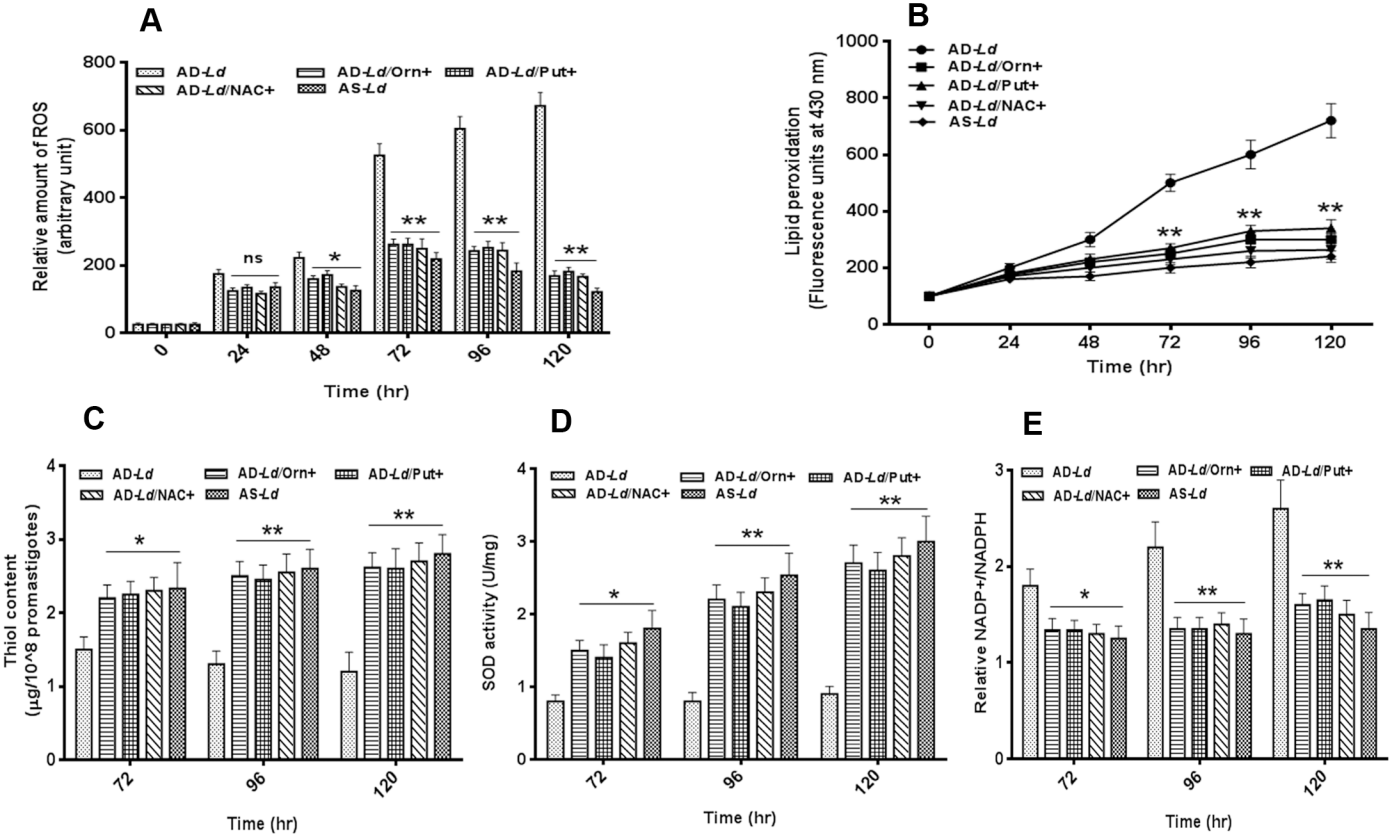
L-arginine availability regulates redox balance in *L*. *donovani* promastigotes. (A-E) *Leishmania* parasites were grown in L-arginine depleted (AD-*Ld*), L-arginine depleted but supplemented with ornithine (AD-*Ld*/Orn+), putrescine (AD-*Ld*/Put+), NAC (AD-*Ld*/NAC+) and L-arginine supplemented (AS-*Ld*) RPMI media for 0–120 hr. Intracellular ROS production was measured using the fluorescent dye 2, 7-dicholorodihydroflourescein diacetate (H_2_DCFDA) and NAC was used as a ROS scavenger (A). The rate of lipid peroxidation was measured by spectrofluorometry and expressed as fluorescence units at 430 nm (B). The total intracellular reduced thiol levels were measured spectrophotometrically using DTNB (C). SOD activity was measured by using SOD assay kit as described in “Materials and methods (D). The relative NADP+/NADPH ratio was measured spectrophotometrically based on the measurement of the absorbance of the reduced coenzyme at 450 nm (E). The data represents mean±SD of triplicate determinations and are representative of three independent experiments.*, P<0.05 (Student’s t test), **, P<0.001 compared to AD-*Ld* or AS-*Ld* parasite as applicable.

The involvement of different forms of ROS including superoxide and hydrogen peroxide (H_2_O_2_) has been reported to play a crucial role in regulation of cellular growth and cell death [50]. In this study, we targeted to explore whether L-arginine deprivation triggered intracellular ROS accumulation and induced apoptosis like cell death in *L*. *donovani* parasites. A gradual increase in ROS level was observed for arginine depleted *Leishmania donovani* (AD-*Ld*) promastigotes (Fig 5A) whereas no significant increase in ROS level was observed for arginine supplemented *Leishmania donovani* (AS-*Ld*) parasites. However, when AD-*Ld* parasites were pre-incubated with NAC, a ROS scavenger, the elevated ROS level fell to the control level. When, we compared the difference in ROS production between AD-*Ld* and AS-*Ld* parasites, it was found that ~2.4 fold, ~3.3 fold and ~3.5 fold induction in ROS level was observed after 72 hr, 96 hr and 120 hr of incubation respectively. Hence, this result indicated that induction in intracellular ROS production might be the possible reason for reduced cell viability for AD-*Ld* parasites.

Further, total fluorescent lipid peroxidised products were spectrophotometrically measured in arginine depleted *Leishmania donovani* (AD-*Ld*) and arginine supplemented *Leishmania donovani* (AS-*Ld*) parasites at 0–120 hr. A gradual significant increase in fluorescence intensity was observed for AD-*Ld* parasites with increasing time of incubation whereas in case of AS-*Ld* parasites, the increase was not significant (Fig 5B).

Non protein thiols are important antioxidants which maintain cellular redox homeostasis through nullifying oxidative perturbations [51]. A gradual decrease in thiol levels was observed for arginine depleted *Leishmania donovani* (AD-*Ld*) parasites with increasing time interval, whereas in case of arginine supplemented *Leishmania donovani* (AS-*Ld*) parasites a slow increase in thiol level was observed. Significant difference (for eg. ~1.6 fold, ~2.0 fold and ~2.3 fold decrease) in thiol content between AD-*Ld* and AS-*Ld* parasites was observed at 72 hr, 96 hr and 120 hr respectively (Fig 5C). The results were consistent with the increased ROS production due to L-arginine deprivation in *Leishmania* parasites.

To determine whether this oxidative stress generated in *L*. *donovani* in case of L-arginine deprivation is associated with reduced antioxidant level, we measured the activity of an anti-oxidant enzyme, SOD, a metal containing enzyme that is present in parasite *L*. *donovani* [52]. ~2.2 fold, ~3.1 fold and ~3.3 fold decrease in SOD activity was observed for arginine depleted *Leishmania donovani* (AD-*Ld*) parasites compared to arginine supplemented *Leishmania donovani* (AS-*Ld*) parasites at 72 hr, 96 hr and 120 hr of incubation respectively (Fig 5D). Therefore, this decrease in SOD activity in AD-*Ld* parasites might be one of the reason for increased ROS production in *Leishmania* parasites and hence decreasing the cell viability.

NADP, an enzymatic cofactor shuttles between the reduced (NADPH) and oxidized (NADP) forms, profoundly supports the major antioxidants and redox regulatory enzymes, by providing reducing potential in the form of NADPH [53]. A gradual increase in relative NADP+/NADPH ratio was observed for arginine depleted *Leishmania donovani* (AD-*Ld*) parasites with increasing time of incubation, whereas no such change was observed for arginine supplemented *Leishmania donovani* (AS-*Ld*) parasites (Fig 5E). A significant difference in relative NADP+/NADPH ratio was observed for AD-*Ld* parasites for 72 hr onwards compared to AS-*Ld* parasites. For eg. ~1.7 fold and ~1.9 fold increase in NADP+/NADPH ratio was observed for AD-*Ld* parasites at 96 hr and 120 hr of incubation. This is correlated with increased ROS production in AD-*Ld* parasites compared to AS-*Ld* parasites.

### *Leishmania* promastigote undergoes an apoptosis-like cell death in absence of L-arginine

**Fig 6:**
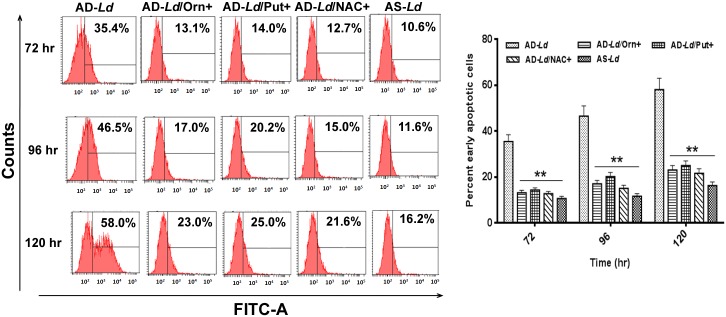
L-arginine deprivation induces externalization of phosphatidyl serine in *Leishmania* parasite. *Leishmania* parasites were grown in L-arginine depleted (AD-*Ld*), L-arginine depleted but supplemented with ornithine (AD-*Ld*/Orn+), putrescine (AD-*Ld*/Put+), NAC (AD-*Ld*/NAC+) and L-arginine supplemented (AS-*Ld*) RPMI media for 72–120 hr. The cells were co-stained with Annexin V-FITC and PI followed by analysis by flow cytometry as described in “Materials and methods”. Histogram of the Annexin-V assay in different conditions as mentioned were shown in figures. The graphs were plotted and percent early apoptotic *Leishmanial* cells were represented. The data represents mean±SD of triplicate determinations and are representative of three independent experiments.*, P<0.05 (Student’s t test)**, P<0.001 compared to AD-*Ld* or AS-*Ld* parasite as applicable.

To elucidate the mechanism of cell death induced by L-arginine deprivation, both arginine depleted *Leishmania donovani* (AD-*Ld*) and arginine supplemented *Leishmania donovani* (AS-*Ld*) promastigotes were stained with Annexin-V-FITC and PI followed by analysis in flow cytometer. Although in both cases, gradual increase in apoptotic cells was observed, the percent of apoptotic cells was higher in case of L-arginine deprivation (Fig 6). Significant differences in percent apoptotic cells were observed at 72 hr, 96 hr and 120 hr (3.3 fold, 4.0 fold and 3.6 fold respectively). However, when AD-*Ld* parasites were supplemented with ornithine (200 mg/L) or putrescine (200 mg/L) or pre-incubated with NAC, a ROS scavenger, the percent apoptotic cells decreased to the control level.

**Fig 7:**
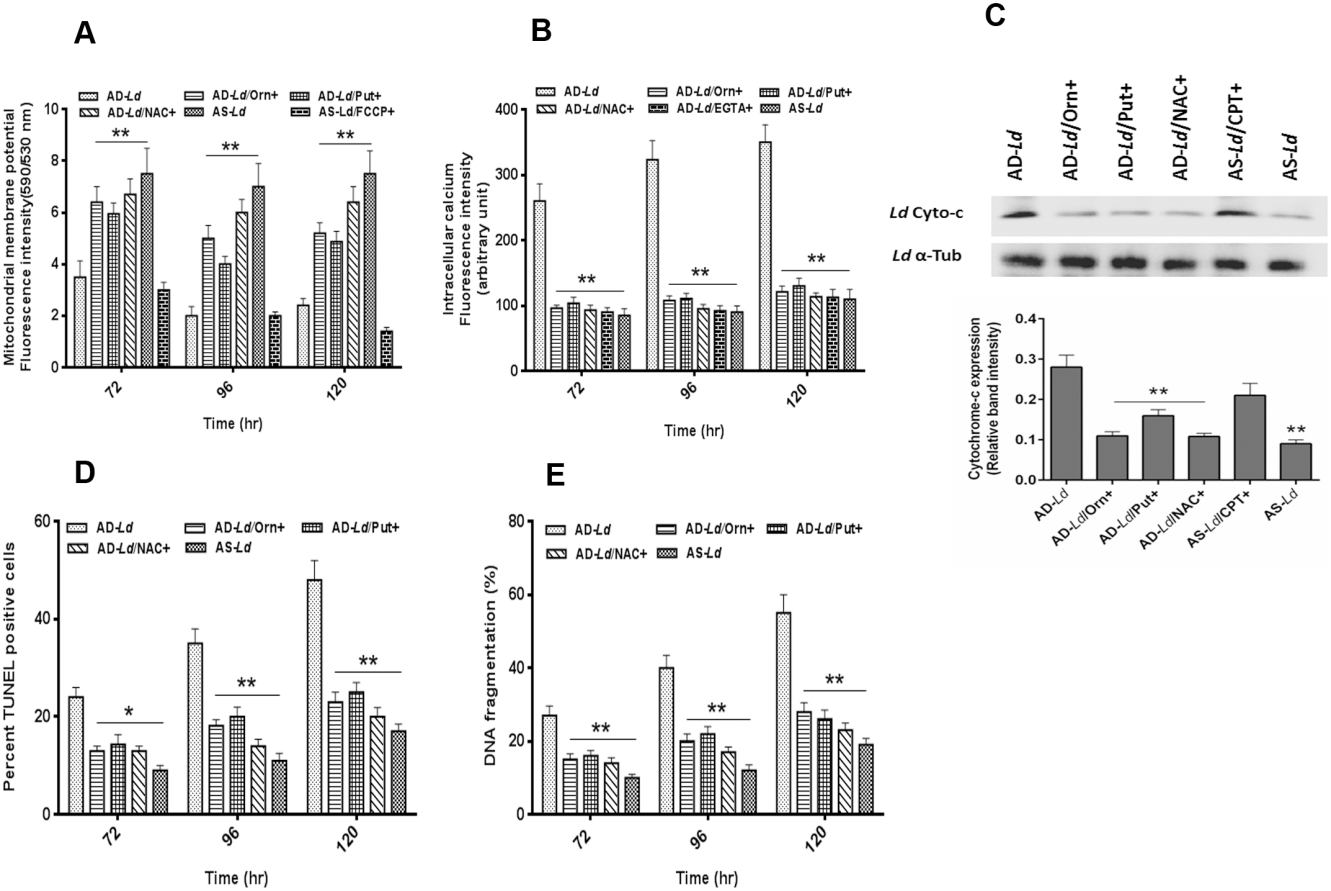
L-arginine deprivation alters mitochondrial membrane potential (Δψm), regulates intracellular Ca^2+^ level, cytochrome-c release and damages DNA in *Leishmania* parasite. (A-E) *Leishmania* parasites were grown in different invitro culture conditions (arginine depletion, AD-*Ld*; arginine depletion but L-ornithine supplementation, AD-*Ld*/Orn+; arginine depletion but putrescine supplementation, AD-*Ld*/Put+; arginine depletion but NAC treatment, AD-*Ld*/NAC+ and arginine supplementation, AS-*Ld*) for 0–120 hr. After individual incubations, the cells were harvested and stained with JC-1 probe and change in mitochondrial membrane potential (Δψm) was determined using spectrofluorometric measurement at 590 and 530 nm. FCCP was used as a positive control for mitochondrial membrane depolarization (A). Intracellular Ca^2+^ concentration was measured by using the fluorescent probe Fura-2-AM followed by spectrofluorometric measurement at 510 nm. EGTA was used as a calcium chelator in this experiment (B). The level of cytochrome-c in cytoplasmic fraction was measured by western blot followed by densitometric analysis and camptothecin-B (CPT) was used as positive control for release of cytochrome-c (C). The occurrence of DNA nicking was detected by the TUNEL assay using flow cytometer as described in “Materials and methods” and percent apoptotic (TUNEL positive) cells were counted (D). DNA fragmentation was measured by cell death detection ELISA (E). The data represents mean±SD of triplicate determinations and are representative of three independent experiments.*, P<0.05 (Student’s t test), **, P<0.001 compared to AD-*Ld* or AS-*Ld* parasite as applicable. ns, non-significant.

Apoptosis in protozoan parasites such as *Leishmania* is characterized by significant fall in mitochondrial membrane potential (Δψm) [10]. JC-1, a cationic lipophilic mito-sensor dye accumulates in the mitochondria of healthy cells. Our assessment of Δψm using JC-1 showed a gradual decrease in fluorescence intensity for arginine depleted *Leishmania donovani* (AD-*Ld*) parasites (Fig 7A). Simultaneously ~2.1 fold, ~3.5 fold and ~3.1 fold decrease in fluorescence intensity was observed for arginine depleted *Leishmania donovani* (AD-*Ld)* parasites when compared to arginine supplemented *Leishmania donovani* (AS-*Ld*) parasites at 72 hr, 96 hr and 120 hr incubation respectively. However, when AD-*Ld* parasites were supplemented with ornithine (200 mg/L) or putrescine (200 mg/L) or pre-incubated with NAC, the fluorescence intensity increased to the control level. Simultaneously, when AS-*Ld* parasites were treated with FCCP, a decrease in fluorescence intensity was observed. This result clearly demonstrated that overproduced ROS in *L*. *donovani* promastigotes led to alterations in mitochondrial membrane potential which validates previous reports [54].

Cytosolic increase in Ca^2+^ level has been found to be associated with mitochondrial membrane depolarization under oxidative stress in *L*. *donovani* [12]. A gradual increase in intracellular calcium concentration was observed for arginine depleted *Leishmania donovani* (AD-*Ld*) parasites whereas in case of arginine supplemented *Leishmania donovani* (AS-*Ld*) parasites no such increase was observed for 0–120 hr. Here, we found ~2.5 fold, ~3.1 fold, ~3.5 fold and ~3.2 fold increase in intracellular calcium level for AD-*Ld* parasites when compared to AS-*Ld* parasites at 48 hr, 72 hr, 96 hr and 120 hr respectively (Fig 7B). However, when AD-*Ld* parasites were supplemented with ornithine (200 mg/L) or putrescine (200 mg/L) or pre-incubated with NAC, the fluorescence intensity decreased to the control level. Furthermore, incubation with extracellular Ca^2+^ chelator, EGTA decreased the intracellular Ca^2+^ to normal level as depicted in the case of AS-*Ld* parasite. Therefore, the above findings mechanistically elucidate that ROS generation due to L-arginine deprivation causes irrepairable damage to calcium channels resulting in enhancement of intracellular calcium reserve followed by loss of Δψm leading to an apoptosis-like cell death.

Cytochrome-c, localized in the inner mitochondrial membrane space, comprises the electron transport chain and is released into the cytosol during the initial steps of apoptosis. We performed western blot hybridization (Fig 7C) to determine the localization of cytochrome-c in arginine depleted *Leishmania donovani* (AD-*Ld*) and arginine supplemented *Leishmania donovani* (AS-*Ld*) parasites. A small amount of cytochrome-c was found in the cytoplasmic fraction of the AS-*Ld* promastigotes whereas cytochrome-c was abundant in the cytoplasmic fraction of AD-*Ld* promastigotes. ~3.1 fold increase in cytochrome-c band intensity was observed for AD-*Ld* parasites compared to AS-*Ld* parasite at 96 hr. However, when AD-*Ld* parasites were supplemented with ornithine (200 mg/L) or putrescine (200 mg/L) or pre-incubated with NAC, significant decrease in release of cytochrome-c compared to AD-*Ld* parasites were observed. Significant increase in release of cytochrome-c was also observed for AS-*Ld* parasites treated with camptothecin (CPT).

Caspase-like protease activity responsive to different apoptotic stimuli has been reported to control the cellular death of unicellular kinetoplastid parasites [10, 24]. Therefore, in the present study, we investigated whether there is any change in caspase-like protease activity due to L-arginine deprivation in *L*. *donovani* parasites. However, we did not find any significant increase in caspase activity (Caspase-1, 3, 5, 8, 9) in arginine depleted *Leishmania donovani* (AD-*Ld*) parasites as compared to arginine supplemented *Leishmania donovani* (AS-*Ld*) parasites (S3A–S3E Fig).

The occurrence of DNA nicking and internucleosomal DNA digestion is a hallmark of apoptosis-like cell death [55]. TUNEL assay based analysis detected FITC labeled dUTP attached to the nick ends through TdT and revealed the presence of nicking of DNA in AD-*Ld* parasites. ~ 2.0 fold, ~2.5 fold and ~2.5 fold increase in percent TUNEL positive cells were observed for arginine depleted *Leishmania donovani* (AD-*Ld*) parasites compared to arginine supplemented *Leishmania donovani* (AS-*Ld*) parasites at 72 hr, 96 hr and 120 hr of incubation respectively (Fig 7D). However, when AD-*Ld* parasites were supplemented with ornithine (200 mg/L) or putrescine (200 mg/L) or pre-incubated with NAC, significant decrease in TUNEL positive cells were observed when compared to AD-*Ld* parasites.

To investigate whether L-arginine deprivation of *L*. *donovani* cells also resulted in occurrence of DNA fragmentation, we carried out genomic DNA fragmentation assay by determining cytoplasmic histone associated mono and oligonucleosomes as described in ‘‘Materials and methods”. ~ 2.7 fold, ~3.3 fold and ~2.9 fold increase in percent DNA fragmentation was observed for arginine depleted *Leishmania donovani* (AD-*Ld*) parasites compared to arginine supplemented *Leishmania donovani* (AS-*Ld*) parasites at 72 hr, 96 hr and 120 hr of incubation respectively (Fig 7E). Furthermore, when AD-*Ld* parasites were supplemented with ornithine (200 mg/L) or putrescine (200 mg/L) or pre-incubated with NAC, significant decrease in percent DNA fragmentation compared to AD-*Ld* parasites were observed.

## Discussion

The importance of L-arginine for the growth of *Leishmania* parasite was demonstrated in 1970s by Krassner et al. [56] indicating that these parasites import this amino acid for the growth in invitro culture media [56, 57]. However, how L-arginine is utilized by the *Leishmania* parasite and promotes growth has not been clearly elucidated.

Our study revealed that the growth of the *Leishmania* parasite is hindered in absence of L-arginine. Simultaneously the rate of proliferation of *Leishmania* parasite also gets reduced in absence of L-arginine, whereas L-arginine supplementation again increases the rate of proliferation, indicating that the growth and proliferation of the *Leishmania* promastigote depends on the availability of L-arginine in the extracellular milieu. Therefore, the decrease in *Leishmania* growth and viability is associated with reduced proliferation. Interestingly supplementation of L-arginine deprived parasite with other amino acids such as lysine, glutamine or proline alone could not restore the growth of the parasite. The survival of the *Leishmania* parasite inside macrophage also gets reduced in presence of inhibitor of arginase. Therefore, it indicates that L-arginine is essential for the growth of the parasite and other amino acids could not act as replacement. This establishes the importance and uniqueness of this amino acid in the survival of the *Leishmania* parasite. Findings from our experiment is in agreement with the previous report [58, 59] which states that as there is no evidence for denovo synthesis of L-arginine in the *Leishmania* parasite, its metabolism depends on extracellular supplies, making L-arginine, an essential amino acid for the growth and survival. Similar type of result was also observed for *T*. *gondii* parasite, an obligate intracellular parasite [60].

L-arginine deprivation also resulted in its increased uptake. However, no difference in uptake of other amino acids such as lysine, glutamine or proline was observed between AD-*Ld* and AS-*Ld* parasites. Our observation is in agreement with the studies of Darlyuk I et al. [61] and indicates that upon sensing low concentration of L-arginine, *L*. *donovani* promastigotes specifically increases the L-arginine uptake probably by upregulating the expression of its transporter.

Polyamines (PAs) like putrescine and spermidine provide critical biosynthetic input in the synthetic machinery of trypanothione, thereby promoting growth and survival of trypanosomatid protozoa [62]. The metabolic process which generates L-ornithine following enzymatic hydrolysis of L-arginine involves arginase on one hand and ODC on the other, to generate putrescine via decarboxylation of L-ornithine. Our previous study suggested that the ROS generated due to autooxidation of amphotericin B was efficiently nullified by polyamine biosynthetic and thiol metabolic pathway enzymes in resistant parasite [35]. Therefore, it is evident that L-ornithine, putrescine along with spermidine feeds the cellular antioxidant machinery to cope up with different forms of oxidative stress. In this study, we found a very low m-RNA expression of polyamine biosynthetic and thiol metabolic pathway genes such as ODC, γ-GCS, SPS and in particular TryS, TR, c-TXN and CTP for L-arginine deprived parasites whereas in case of L-arginine supplemented parasites all these genes were upregulated. The activity of arginase, the first enzyme of arginine metabolism was also found to be downregulated for L-arginine deprived parasites. The activity of the enzyme depends on the availability of the substrate. Here, as the substrate, L-arginine is limited; the activity of the downstream enzyme gets decreased both at transcript and translational level as observed through semi-quantitative RT-PCR and immunoblot analysis respectively. It can therefore be concluded that due to non-availability of arginine, the expression of polyamine biosynthetic and thiol metabolic pathway enzymes get downregulated and that results in the decreased production of polyamines such as putrescine and spermidine.

To understand the mechanism of reduced growth rate and cell viability of *L*. *donovani* parasite in L-arginine depleted media, we assayed intracellular ROS generation. Interestingly, significant increase in intracellular ROS generation was observed for AD-*Ld* parasites compared to AS-*Ld* parasites. It was also observed that L-arginine supplementation decreases the ROS level. Induction of ROS due to nutrient deprivation (such as glucose and amino acid) has been reported previously [63]. To the best of our knowledge this is the first report describing ROS generation due to L-arginine deprivation in *L*. *donovani*. Therefore, it can be concluded that ROS generation might be one of the causes for reduced cell viability and growth of *Leishmania* parasite in L-arginine depleted media.

Studies of Das et al. [64] showed that curcumin treatment to *Leishmania* parasite leads to an increase in lipid peroxides levels. Increase in lipid peroxide with increasing time was also observed for *L*. *donovani* after spiningerin treatment by Sardar et al. [43]. In this study, we found a significant increase in lipid peroxide level for *Leishmania* promastigotes grown in L-arginine depleted media with increasing time interval, whereas L-arginine supplementation restores this increase in lipid peroxidation. Hence, it can be concluded that ROS generation due to L-arginine deprivation might be one of the causes for increasing lipid peroxidation that ultimately leads to reduced cell viability and growth of *Leishmania* parasite.

The free radicals elicited by the phagocytic macrophages is successfully evaded by the parasitic protozoan *Leishmania* via utilization of elaborate network of non-protein thiols like glutathione and trypanothione [51]. In this study, a significant difference in thiol level between AD-*Ld* and AS-*Ld* parasite was observed with increasing time of incubation. The decreased intracellular thiol level in AD-*Ld* parasites is correlated with increased intracellular ROS production leading to decreased parasite survival.

The cellular concentration of ROS is controlled by anti-oxidant enzymes. The balance between ROS generation and ROS elimination by antioxidant enzymes helps to maintain cellular function. Therefore, a decrease in anti-oxidant enzyme levels leads to an overall increase in intracellular ROS levels and causes cell death [65]. The involvement of SOD in detoxification of reactive superoxide radicals produced by activated macrophages and survival had been described in *Leishmania* promastigotes [66]. In this study, we tried to investigate the probable reason of reduced cell viability of *Leishmania donovani* in L-arginine deprived condition. As increased ROS level is associated with reduced cell survival and apoptosis and SOD is an anti-oxidant enzyme that helps to neutralize ROS, we measured the activity of SOD. In this study, a gradual decrease in SOD activity was observed for *Leishmania* parasites grown in L-arginine deprived media, whereas in case of parasites grown in L-arginine enriched media, SOD activity gradually increased. Therefore, it is obvious that this reduced SOD activity led to increased ROS generation in L-arginine deprived condition. Finally, we concluded that the reduction in cell viability of AD-*Ld* parasite was due to increased ROS generation and decreased thiol levels.

NADP+/NADPH ratio critically regulates the oxidative homeostasis inside cell. In this study an increased NADP+/NADPH ratio was observed for L-arginine deprived parasites with increasing time interval which further supports our hypothesis of ROS production due to L-arginine deprivation resulting in reduced viability of *Leishmania* parasite.

Apoptosis-like phenomenon was observed in *L*. *donovani* promastigotes upon treatment with hydrogen peroxide [10, 12] and nutrient deprivation [24] whereas staurosporine treatment [67] and serum withdrawal [18] demonstrated similar changes in case of *L*. *major* promastigotes. To the best of our knowledge, this is the first report where we showed significant increase in early apoptotic cells in L-arginine deprivation condition with increasing time of incubation.

**Fig 8:**
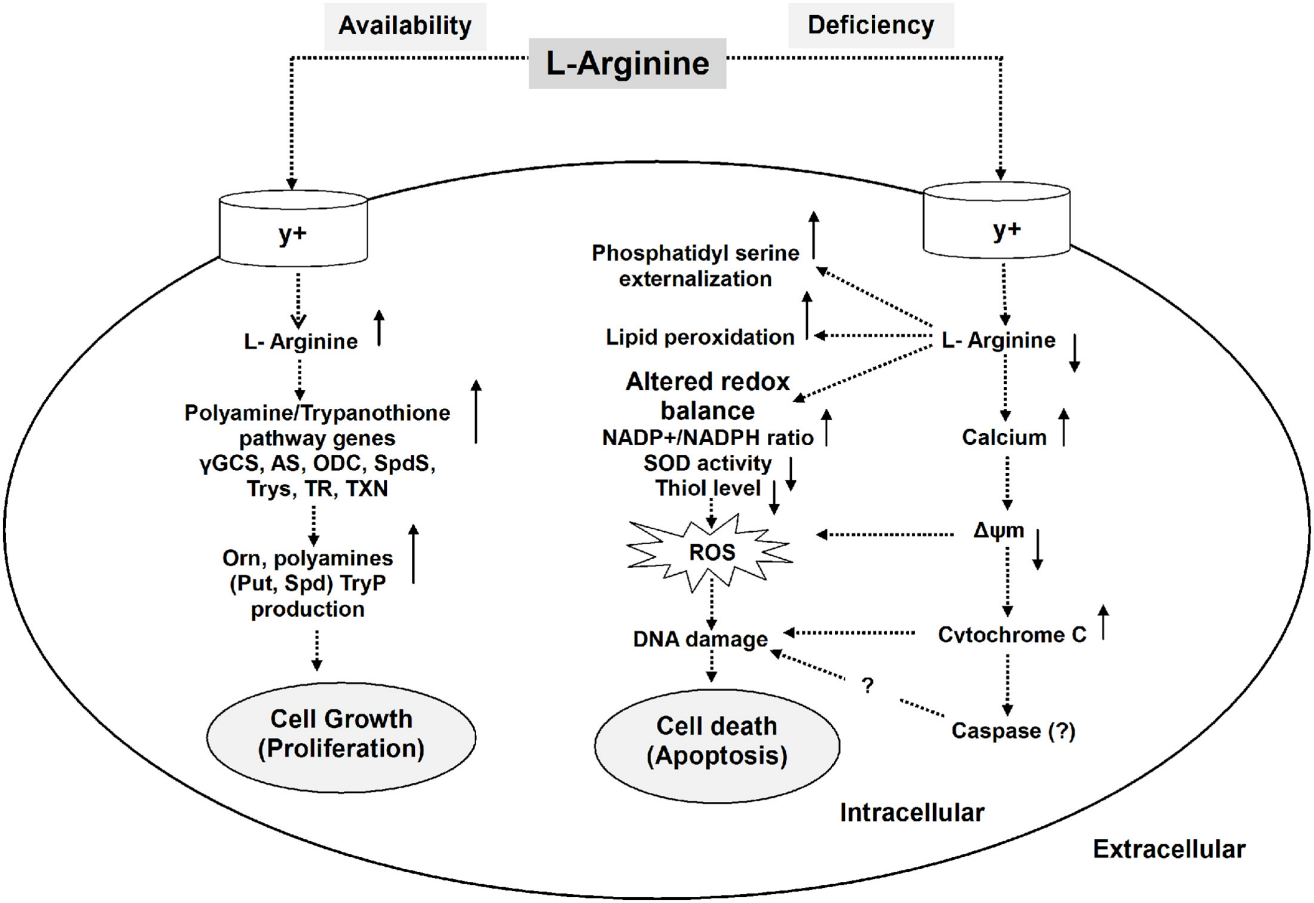
Schematic representation demonstrating the role of L-arginine in the modulation of cell proliferation or apoptosis-like cell death in *Leishmania* parasite. The model illustrates that the fate of *Leishmania* parasites depend on the availability of extracellular L-arginine. If L-arginine is available, it is taken up from the external medium by cationic L-arginine transporters (y+) and metabolized by arginase (AS) and other polyamine biosynthetic and thiol metabolic pathway enzymes such as γ-glutamylcysteine synthetase (γ-GCS), ornithine decarboxylase (ODC), spermidine synthase (SpdS), trypanothione synthetase (TryS), trypanothione reductase (TR), tryparedoxin (TXN) to produce L-ornithine (Orn), putrescine (Put), spermidine (Spd) and thiols such as trypanothione (TryP) which promotes parasite proliferation and cell growth. Whereas, if extracellular L-arginine is unavailable or deficient, cellular redox balance is altered characterized by increased NADP+/NADPH ratio, decreased antioxidant levels such as SOD activity and thiol content that led to increased ROS production. L-arginine unavailability also triggered the phenomenons like phosphatidyl serine externalization, increased lipid peroxidation and release of intracellular calcium. Calcium influx in turn causes mitochondrial membrane depolarization leading to release of cytochrome-c from mitochondrion and intracellular ROS generation that ultimately damages DNA and promotes an apoptosis-like cell death.

When we measured the alteration in mitochondrial membrane potential (Δψm), we found that as expected with increasing time interval, a significant decrease in Δψm was observed for AD-*Ld* parasites compared to AS-*Ld* parasites. High ROS levels bring about cationic influx in cytosol which leads to mitochondrial dysfunction and depolarization [68]. With already established evidences of calcium influx modulating the activation of different proteases and regulating the exhibition of phosphatidyl serine on the outer leaflet of plasma membrane during apoptosis [69], we considered to measure the Ca^2+^ concentration in L-arginine deprived and L-arginine supplemented *Leishmania* parasites. Interestingly, a gradual increase in intracellular cytosolic Ca^2+^ level was observed for AD-*Ld* parasites whereas no such change was observed for AS-*Ld* parasites. Simultaneously, significant increase in cytosolic cytochrome-c was also observed for AD-*Ld* parasite compared to AS-*Ld* parasite. Therefore, the increase in cytosolic Ca^2+^ and cytochrome-c is correlated with increased ROS level resulting in decrased cell viability in L-arginine deprivation condition. Collectively, our results clearly suggest that the biochemical basis behind reduced L-arginine availability and increased ROS generation is due to downregulation of SOD activity, increased NADP+/NADPH ratio, reduced thiol level, release of intracellular calcium and decreased mitochondrial membrane potential (Fig 8).

As the apoptotic pathways are stringently regulated by caspases, the presence of caspase-like activity in the parasites under L-arginine deprivation condition was examined. However, recent apoptosis work challenges the accepted roles of caspase proteases [23]. Dolai et al. [70] also reported ROS dependent but caspase independent apoptosis-like cell death in *L*. *major* promastigotes. Interestingly in our study, no significant increase in caspase activity was observed for AD-*Ld* parasites compared to AS-*Ld* parasites with increasing time intervals.

To further confirm apoptosis-like cell death of *Leishmania* parasite, we analyzed DNA nicking by TUNEL assay using flow cytometer and DNA fragmentation by Cell Death Detection ELISA. As expected, a significant increase in TUNEL positive cells and DNA fragmentation was observed for AD-*Ld* parasites compared to AS-*Ld* parasites with increasing time intervals.

From this study, it can be concluded that L-arginine is a crucial molecule for survival and growth of *Leishmania* parasite, in absence of which the parasite undergoes an apoptosis-like cell death. Our study is the first report describing the mechanism of L-arginine dependent regulation of cell survival and apoptosis in *L*. *donovani* promastigotes (Fig 8). The molecular mechanism for L-arginine regulated caspase independent apoptosis-like cell death involves ROS mediated DNA degradation through intracellular calcium influx and subsequent depolarization of mitochondrial membrane potential along with cytosolic cytochrome-c release. Therefore, this important amino acid might be targeted for the efficient control of *Leishmania* growth and thereby occurrence of VL.

## Supporting Information

S1 Fig L-arginine deprivation has a marked effect in reduction of cell viability of *Leishmania* parasite compared to other amino acids. (A) *L*. *donovani* promastigotes were grown in amino acid free (AA-), L-arginine free (Arg-), L-lysine free (Lys-), L-glutamine free (Gln-), L-proline free (Pro-) and normal (ctrl) RPMI media separately for 0–120 hr. The percent cell viability of the parasite was determined by MTT assay. (B) *L*. *donovani* promastigotes were grown in amino acid free (AA-) as well as amino acid free but supplemented with individual amino acids such as L-arginine (AA-/Arg+), L-lysine (AA-/Lys+), L-glutamine (AA-/Gln+), L-proline (AA-/Pro+) and normal (ctrl) RPMI separately for 0–120 hr. The percent cell viability of the parasite was determined by MTT assay. The data represents mean±SD of triplicate determinations and are representative of three independent experiments. *, P<0.05 (Student’s t test), **, P<0.001 compared to control (or AA- as applicable). ns, non-significant
(TIF)

S2 Fig Concentration kinetics of radioactive L-arginine transport in *Leishmania* parasite. *Leishmania* parasites were grown in L-arginine depleted (AD-*Ld*) and L-arginine supplemented (AS-*Ld*) RPMI media. After 96 hr of incubation the parasites were harvested, incubated with different conc. of [^3^H] L-arginine (0–5 μCi/ml) for 8 minutes and rate of uptake was measured. The data represents mean±SD of triplicate determinations and are representative of three independent experiments.*, P<0.05 (Student’s t test), **, P<0.001 compared to AD-*Ld* or AS-*Ld* parasite as applicable. ns, non-significant.
(TIF)

S3 Fig L-arginine deprivation had no effect on caspase like protease activities in *Leishmania* parasites. (A-E) *Leishmania* parasites were grown in L-arginine depleted (AD-*Ld*) and L-arginine supplemented (AS-*Ld*) RPMI media for 0–120 hr. The activity of different caspases [caspase-1 (A), caspase-3 (B), caspase-5 (C), caspase-8 (D) and caspase-9 (E)] were measured using a fluorogenic homogeneous caspase assay kit as described in “Materials and methods”. The data represents mean±SD of triplicate determinations and are representative of three independent experiments.*, P<0.05 (Student’s t test), **, P<0.001 compared to AD-*Ld* or AS-*Ld* parasite as applicable. ns, non-significant.
(TIF)

S1 Table Primers used for semiQ-RT PCR reactions. (DOCX)
