# Expression of Concern: Deprivation of L-Arginine Induces Oxidative Stress Mediated Apoptosis in *Leishmania donovani* Promastigotes: Contribution of the Polyamine Pathway

**DOI:** 10.1371/journal.pntd.0009580

**Published:** 2021-07-08

**Authors:** The PLOS Neglected Tropical Diseases Editors

Following the publication of this article [1], concerns were raised regarding results presented in Fig 6. Specifically,

- The results presented for the 72 hr AD-*Ld/*Orn+, 72 hr AD-*Ld/*Put+, 72 hr AD-*Ld/*NAC+, and 96 hr AD-*Ld/*NAC+ appear similar but not quite identical.
- The results presented for the 120 hr AD-*Ld/*Orn+ and 120 AD-*Ld/*Put+ results appear similar but not quite identical.

The authors submitted higher resolution versions of the panels presented in Fig 6, provided in the S1 File below. A member of the *PLOS Neglected Tropical Diseases* board assessed the concerns and the data provided by the author and indicated that the higher resolution images highlight minor differences between the 72 hr AD-*Ld/*Orn+, 72 hr AD-*Ld/*Put+, 72 hr AD-*Ld/*NAC+, and 96 hr AD-*Ld/*NAC+ panels, but that the 120 hr AD-*Ld/*Orn+ and 120 AD-*Ld/*Put+ panels appear more similar than would be expected from independent samples.

The raw flow cytometry data underlying the panels presented in Fig 6 are no longer available. In the absence of the original data underlying Fig 6, PLOS *Neglected Tropical Diseases* cannot confirm the reliability of the results presented in this figure.

The raw data and individual level data underlying the other results presented in this article are no longer available.

The PLOS *Neglected Tropical Diseases* Editors issue this Expression of Concern to notify readers of the above concerns and relay the higher resolution figures provided by the corresponding author.

## Supporting information

S1 File Higher resolution copies of the panels presented in Fig 6. (RAR)
