# Supplementary figures and images for: Deprivation of L-Arginine Induces Oxidative Stress Mediated Apoptosis in Leishmania donovani Promastigotes: Contribution of the Polyamine Pathway
Source: PLoS Negl Trop Dis. 2016 Jan 25;10(1):e0004373. doi: 10.1371/journal.pntd.0004373 (PMC4726550; doi:10.1371/journal.pntd.0004373)

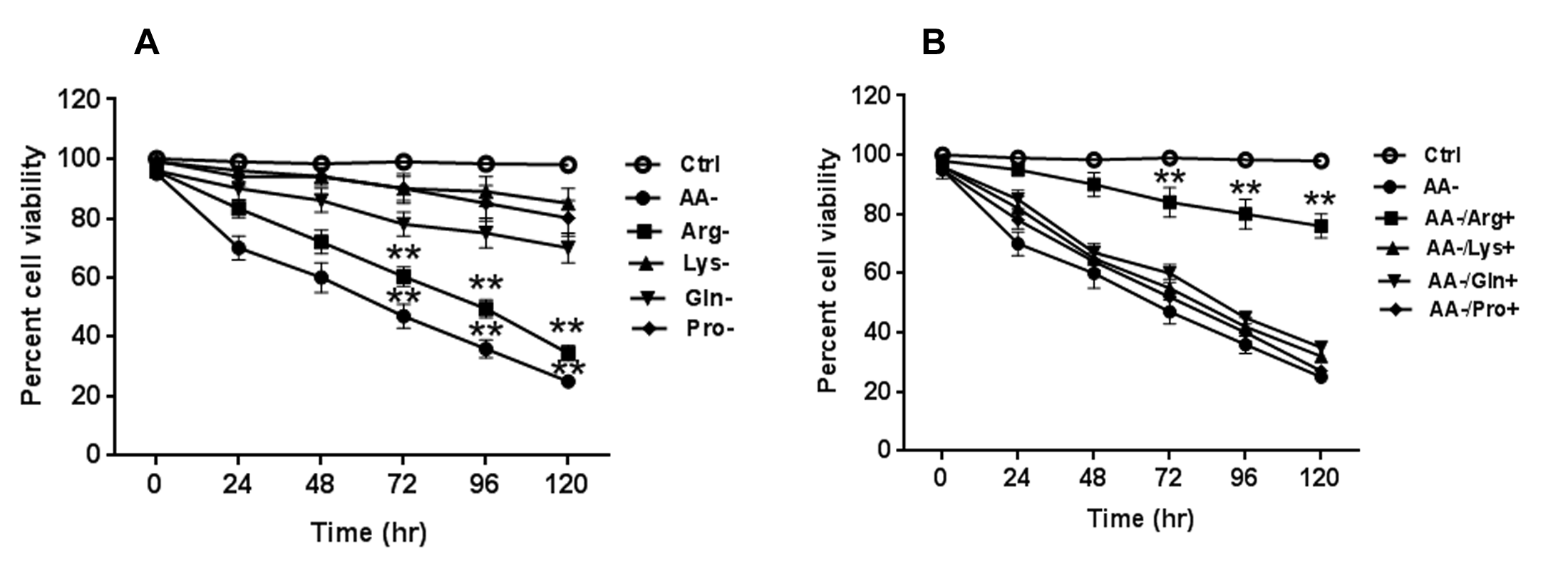

Supplement: S1 Fig — (A) L. donovani promastigotes were grown in amino acid free (AA-), L-arginine free (Arg-), L-lysine free (Lys-), L-glutamine free (Gln-), L-proline free (Pro-) and normal (ctrl) RPMI media separately for 0–120 hr. The percent cell viability of the parasite was determined by MTT assay. (B) L. donovani promastigotes were grown in amino acid free (AA-) as well as amino acid free but supplemented with individual amino acids such as L-arginine (AA-/Arg+), L-lysine (AA-/Lys+), L-glutamine (AA-/Gln+), L-proline (AA-/Pro+) and normal (ctrl) RPMI separately for 0–120 hr. The percent cell viability of the parasite was determined by MTT assay. The data represents mean±SD of triplicate determinations and are representative of three independent experiments. *, P<0.05 (Student’s t test), **, P<0.001 compared to control (or AA- as applicable). ns, non-significant (TIF) [file pntd.0004373.s001.tif]

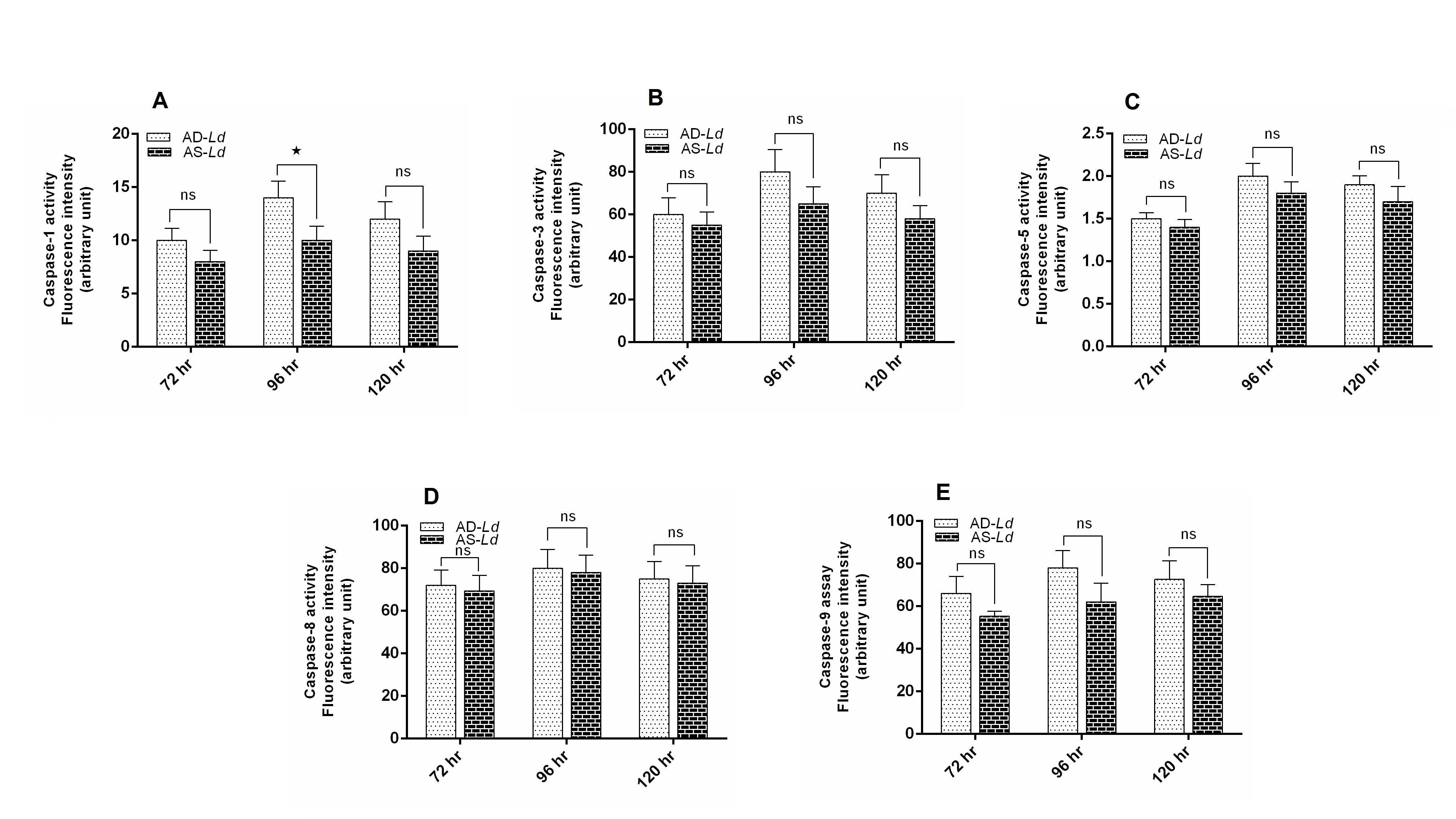

Supplement: S3 Fig — (A-E) Leishmania parasites were grown in L-arginine depleted (AD-Ld) and L-arginine supplemented (AS-Ld) RPMI media for 0–120 hr. The activity of different caspases [caspase-1 (A), caspase-3 (B), caspase-5 (C), caspase-8 (D) and caspase-9 (E)] were measured using a fluorogenic homogeneous caspase assay kit as described in “Materials and methods”. The data represents mean±SD of triplicate determinations and are representative of three independent experiments.*, P<0.05 (Student’s t test), **, P<0.001 compared to AD-Ld or AS-Ld parasite as applicable. ns, non-significant. (TIF) [file pntd.0004373.s003.tif]
