# Supplementary material for: Deprivation of L-Arginine Induces Oxidative Stress Mediated Apoptosis in Leishmania donovani Promastigotes: Contribution of the Polyamine Pathway
Source: PLoS Negl Trop Dis. 2016 Jan 25;10(1):e0004373. doi: 10.1371/journal.pntd.0004373 (PMC4726550; doi:10.1371/journal.pntd.0004373)
Supplement: S1 Table — (DOCX) [file pntd.0004373.s004.docx]

**Supplemental material**

**Table S1**. Primers used for semiQ-RT PCR reactions

| **Gene** | **Forward primer** | **Reverse primer** |
| --- | --- | --- |
| ODC | 5’-GCACGCGCTTCTCATGAACGTATT-3’ | 5’-CGAAGAGGATGCAGTTGAAGCTGT-3’ |
| SPS | 5’-ACTTCTACACGAATGTGCTCCGCA-3’ | 5’-CATTGCGTACTTGACCGTGGCAAA-3’ |
| γ-GCS | 5’-AGCGATAAACCGCTCGTACTGTGA-3’ | 5’-ATGTTGTCAAAGTGCTCCGTGTGC-3’ |
| TryS | 5’-TGTCATGAGCGAATGACCAACCGAT-3’ | 5’-GCTTGCCATTCAACAAACGTCAGGT-3’ |
| TR | 5’-AATGAGGACGGCTCGAATCACGTT-3’ | 5’-ATGGCGTAGATGTTGTCCACCGAT-3’ |
| c-TXN | 5’-AAGCTAAACACGCAGGTTGTTGCG-3’ | 5’-ATACCGGATTCCTCGATCAGCACA-3’ |
| CTP | 5’-CCAACGGCAGCTTCAAGAAGATCA-3’ | 5’-TGAAGTCGAGCGGGTAGAAGAAGA-3’ |
